# Manipulation of Glutamatergic Neuronal Activity in the Primary Motor Cortex Regulates Cardiac Function in Normal and Myocardial Infarction Mice

**DOI:** 10.1002/advs.202305581

**Published:** 2024-03-15

**Authors:** Wenyan Bo, Mengxin Cai, Yixuan Ma, Lingyun Di, Yanbin Geng, Hangzhuo Li, Caicai Tang, Fadao Tai, Zhixiong He, Zhenjun Tian

**Affiliations:** ^1^ Institute of Sports and Exercise Biology, Institute of Brain and Behavioral Sciences Shaanxi Normal University Xi'an 710119 China

**Keywords:** cardiac function, median raphe nuclei, myocardial infarction, primary motor cortex

## Abstract

Cardiac function is under neural regulation; however, brain regions in the cerebral cortex responsible for regulating cardiac function remain elusive. In this study, retrograde trans‐synaptic viral tracing is used from the heart to identify a specific population of the excitatory neurons in the primary motor cortex (M1) that influences cardiac function in mice. Optogenetic activation of M1 glutamatergic neurons increases heart rate, ejection fraction, and blood pressure. By contrast, inhibition of M1 glutamatergic neurons decreased cardiac function and blood pressure as well as tyrosine hydroxylase (TH) expression in the heart. Using viral tracing and optogenetics, the median raphe nucleus (MnR) is identified as one of the key relay brain regions in the circuit from M1 that affect cardiac function. Then, a mouse model of cardiac injury is established caused by myocardial infarction (MI), in which optogenetic activation of M1 glutamatergic neurons impaired cardiac function in MI mice. Moreover, ablation of M1 neurons decreased the levels of norepinephrine and cardiac TH expression, and enhanced cardiac function in MI mice. These findings establish that the M1 neurons involved in the regulation of cardiac function and blood pressure. They also help the understanding of the neural mechanisms underlying cardiovascular regulation.

## Graphical abstract

1.Trans‐synaptic viral tracing from the heart identifies a cluster of the M1 pyramidal cells. 2.Manipulation of M1 glutamatergic neurons affects cardiac function and blood pressure in normal mice and mice with myocardial infarction through projecting to MnR. These results offer new ideas and targets for studying the pathogenesis and treatment of brain‐heart syndrome.

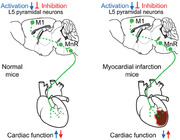

## 1 Introduction

It is well‐known that the activity and function of the heart are controlled by a complex neural network involving certain higher cortical areas, including the orbitofrontal cortex, the dorsal cingulate cortex, the prefrontal cortex, the insula, and the subcortical forebrain areas including the amygdala and the hippocampus, the hypothalamus, the bed nucleus of the stria terminalis, the periaqueductal gray, the parabrachial region, and the ventrolateral medulla.^[^ ^1^ ^]^ However, the exact brain regions in the cerebral cortex that influence heart function are still not fully clarified.

In a recent review, Mohanta et al. highlighted two majors neural subcircuits regulating the cardiovascular system: the artery‐brain circuit and the heart‐brain circuit. The major components in the heart‐brain circuit includes the sensory arm composed of vagus afferents and spinal nerves from the heart, the cardiovascular center in the brainstem and higher brain, parasympathetic and sympathetic efferent nerves, and local neural networks in the heart.^[^ ^2^ ^]^ It has been reported that the heart receives projections from the prelimbic, anterior cingulate, frontal cortex, and insular cortex.^[^ ^3^ ^]^ Stimulation of the insular cortex resulted in an increase in atrioventricular conduction, alteration of heart rate and cardiac injury.^[^ ^4^ ^]^ This is in association with the insular cortex's regulation of emotional heart activity.^[^ ^5^ ^]^ By contrast, cortex damage, such as traumatic brain injury, can lead to cardiac injury and dysfunction as well as the increased neuronal apoptosis in the peri‐contusion cortex.^[^ ^6^ ^]^ These results indicate that the cortical areas are involved in the regulation of cardiac function. However, the exact location and the identity of the cortical neurons that influence cardiac function have not been fully elucidated.

The primary motor cortex (M1) is a well‐known pre‐dominator of motor execution, and functions in higher cognitive processes, such as attention, motor learning and consolidation, movement inhibition, somatomotor response, and movement imagery.^[^ ^7^ ^]^ Recent studies have reported that the M1 region is involved in pain modulation.^[^ ^8^ ^]^ It is worth noting that Li et al. and our preliminary experiment have identified the connection between M1 region and heart using pseudorabies virus (PRV) as a tracer.^[^ ^9^ ^]^ Although M1 neurons project to multiple brain regions, the circuits related to cardiac function remain unclear.^[^ ^10^ ^]^ In addition, while many studies focused on the neuroimmune axis of cardiovascular control and the function in regulating atherosclerosis,^[^ ^11^ ^]^ little is known about the regulatory function of M1 on injured heart. Thus, clarification of the influence of M1‐heart connection on injured heart is also required in understanding the functions of this cortex‐heart circuit.

To answer these questions, we studied the effects of modulating M1 activity on cardiac function and blood pressure in mice through a combination of several approaches including virus‐based tracing, optogenetics, chemogenetics, induced‐neuronal apoptosis, and in vivo neuronal activity recording. We also established a myocardial infarction (MI) model in mice and observed effects of M1 neuronal activities on heart function. This study provides important evidence for in‐depth analysis of the exact relationship between the brain and heart, and offer new targets for studying the pathogenesis and treatment of brain‐heart syndrome.

## 2 Results

### 2.1 Identification of a Cluster of the M1 Pyramidal Cells through PRV Tracing from the Heart

**Figure 1:**
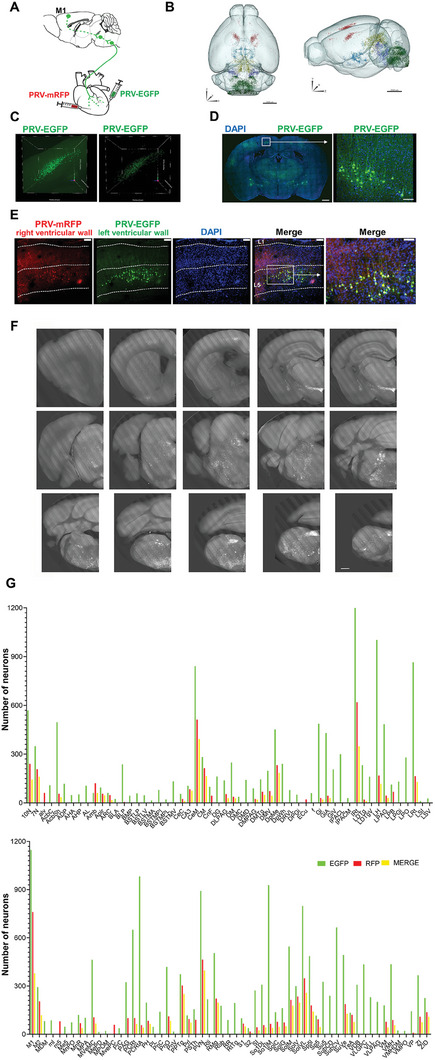
A perspective on whole‐brain imaging through cardiac‐derived pseudorabies virus tracing. A) PRV‐EGFP or PRV‐mRFP was respectively injected into the left or right ventricular wall. B) Sagittal (left) and horizontal (right) views of PRV‐positive neurons in the whole brain of mouse, including red (cortex), blue (hypothalamus), yellow (superior colliculus), violet (midbrain), and green (pons, medulla, spinal cord). C) Representative 3D reconstruction of PRV‐EGFP labeled cortical neurons. D) Representative images showing EGFP‐positive neurons (green) in the cortex. Scale bar, 500 µm (left) and 100 µm (right). E) Representative coronal brain sections showing co‐labeling of mRFP and EGFP‐positive neurons in M1 and S1 from the same mouse 5.5 days after receiving PRV‐EGFP injection into the left ventricular wall and PRV‐mRFP injection into the right ventricular wall 5.5 days previously. Scale bar, 88.52 µm. F) Images are representative of positively labeled neurons in the brain. Scale bar, 500 µm. G) The number of positive neurons in the brain. Abbreviations are: 10N, dorsal motor nucleus of vagus;7N, facial nucleus; alv, alveus of the hippocampus; AcbC, accumbent nucleus core; AcbSh, accumbent nucleus shell; ADP, anterodorsal preoptic nucleus; AHA, anterior hypothalamic area, anterior part; AHP, anterior hypothalamic area, posterior part; AL, nucleus of the ansa lenticularis; Amb, ambiguous nucleus; Apir, amygdalopiriform transition area; ARC, arcuate hypothalamic nucleus; BLA, basolateral amygdaloid nucleus anterior part; BLP, basolateral amygdaloid nucleus posterior part; BMP, basomedial amygdaloid nucleus posterior part; BSTLP, bed nucleus of the stria terminalis, lateral division posterior part; BSTLV, bed nucleus of the stria terminalis, lateral division ventral part; BSTMA, bed nucleus of the stria terminalis, medial division anterior part; BSTMPI, bed nucleus of the stria terminalis, medial division posterolateral part; BSTMPL, bed nucleus of the stria terminalis, medial division, posterolateral part; BSTMV, bed nucleus of the stria terminalis, medial division ventral part; CeC, central amygdaloid nucleus, capsular part; CA3, field CA3 of hippocampus; CeM, central amygdaloid nucleus, medial division; CnF, cuneiform nucleus; DG, dentate gyrus; DLPAG, dorsolateral periaqueductal gray; DM, dorsomedial hypothalamic nucleus; DMC, dorsomedial hypothalamic nucleus compact part; DMD, dorsomedial hypothalamic nucleus dorsal part; DMPAG, dorsomedial periaqueductal gray; DMTg, dorsomedial tegmental area; DMV, dorsomedial hypothalamic nucleus ventral part; DpMe, deep mesencephalic nucleus; DpWh, deep white layer of the superior colliculus; DRVL, dorsal raphe nucleus, ventrolateral part; DPGi, dorsal paragigantocellular nucleus; ECu, external cuneate nucleus; f, fornix; Gi, gigantocellular reticular nucleus; GiA, gigantocellular reticular nucleus alpha part; GiV, gigantocellular reticular nucleus, ventral part; IPACL, interstitial nucleus of the posterior limb of the anterior commissure, later part; IPACM, interstitial nnucleus of the posterior limb of the anterior commissure, medial part; IRt, intermediate reticular nucleus; LDTg, laterodorsal tegmental nucleus; LDTgV, laterodorsal tegmental nucleus ventral part; LH, lateral hypothalamic area; LPAG, lateral periaueductal gray; LPB, lateral parabrachial nucleus; LPGi, lateral paragigantocellular nucleus; LPO, lateral preoptic area; LRt, lateral reticular nucleus; LSI, lateral septal nucleus, intermediate part; LSV, lateral septal nucleus, ventral part; M1, primary motor cortex; M2, secondary motor cortex; MDM, mediodorsal thalamic nucleus, medial part; ml, medial lemniscus; m5, motor root of the trigeminal nerve; Me5, mesencephalic trigeminal nucleus; MnPO, median preoptic nucleus; MnR, median raphe nucleus; MPA, medial preoptic area; MVeMC, medial vestibular nucleus, magnocellular part; MePD, medial amygdaloid nucleus, posterodorsal part; MPOM, medial preoptic nucleus, medial part; MvePC, medial vestibular nucleus, parvicellular part; PrC, precommissural nucleus; PAG, periaqueductal gray; PCRt, parvicellular reticular nucleus; PCRtA, parvicellular reticular nucleus, alpha part; PH, posterior hypothalamic area; PL, paralemniscal nucleus; PnC, pontine reticular nucleus, caudal part; PnO, pontine reticular nucleus, oral part; PnV, pontine reticular nucleus, ventral part; PPTg, pedunculopontine tegmental nucleus; Prl, prelimbic cortex; PSTh, parasubthalamic nucleus; PVN, paraventricular hypothalamic nucleus; Rli, rostral linear nucleus of the raphe; RMg, raphe magnus nucleus; Rob, raphe of obscurus nucleus; RR, retrorubral nucleus; RtTg, reticulotegmental nucleus of the pons; S1, primary somatosensory cortex; S2, secondary somatosensory cortex; SI, substantia innominate; So1DL, solitary tract, dorsolateral part; So1lM, nucleus of the solitary tract, intermediate part; SolC, nucleus of the solitary tract, commissural part; SolG, nucleus of the solitary tract, gelatinous part; SolM, nucleus of the solitary tract, medial part; SolV, solitary tract, ventral part; SolVL, nucleus of the solitary tract, ventrolateral part; Sp5l, spinal trigeminal tract, interpolar part; Sp5, spinal trigeminal tract; Su5, supratrigeminal nucleus; SubCD, subcoeruleus dorsal nucleus; SubCV, subcoeruleus nucleus, ventral part; SuVe, superior vestibular nucleus; TeA, temporal association cortex; VDB, nucleus of the vertical limb of the diagonal band; VLGPC, ventral lateral geniculate nucleus, parvicellular part; VLL, ventral nucleus of the lateral lemniscus; VLPAG, ventrolateral periaqueductal gray; VM, ventromedial thalamic nucleus; VMH, ventromedial hypothalamic nucleus; VMHDM, ventromedial hypothalamic nucleus, dorsomedial part; VMPO, ventromedial preoptic nucleus; VP, ventral pallidum; ZI, zona incerta; ZID, zona incerta, dorsal part.

To identify candidate brain regions that potentially regulate heart function, we injected PRV‐EGFP or PRV‐mRFP into the left or right ventricular wall respectively (**Figure** **1**A), as described in previous studies.^[^ ^12^ ^]^ Given that PRV clearly labeled neurons in brain, we reconstructed the mouse brain by fluorescence Micro‐Optical Sectioning Tomography (fMOST) (Figure 1B and Video S1, Supporting Information). Robust EGFP/mCherry labeling was detected in many brain regions with well‐known projections to the heart after 5.5 days, including the brainstem MnR, the nucleus of the solitary tract (NTS) and the bilateral rostral ventrolateral medulla (RVLM), the hypothalamus (medial preoptic area: MPOA), the paraventricular nucleus (PVN), the ventromedial hypothalamus (VMH), and the medial prefrontal cortex (mPFC) (Figure 1F,G, n = 3). There were no labeled neurons in M1, PVN and other brain regions at the day 3 (Figure S1, Supporting Information). Unexpectedly, PRV‐labeled neurons were also found in M1, secondary motor cortex (M2) and somatosensory cortex (Figure 1C,D,E). Interestingly, infected cortical neurons showed ∼40% overlap of EGFP and RFP in the M1 region, suggesting that neurons in the M1 region may simultaneously control myocardial activity in the left and right ventricles (Figure 1E).

### 2.2 Manipulation of M1 Glutamatergic Neurons Modulated Cardiac Function and Blood Pressure in Normal Mice

**Figure 2:**
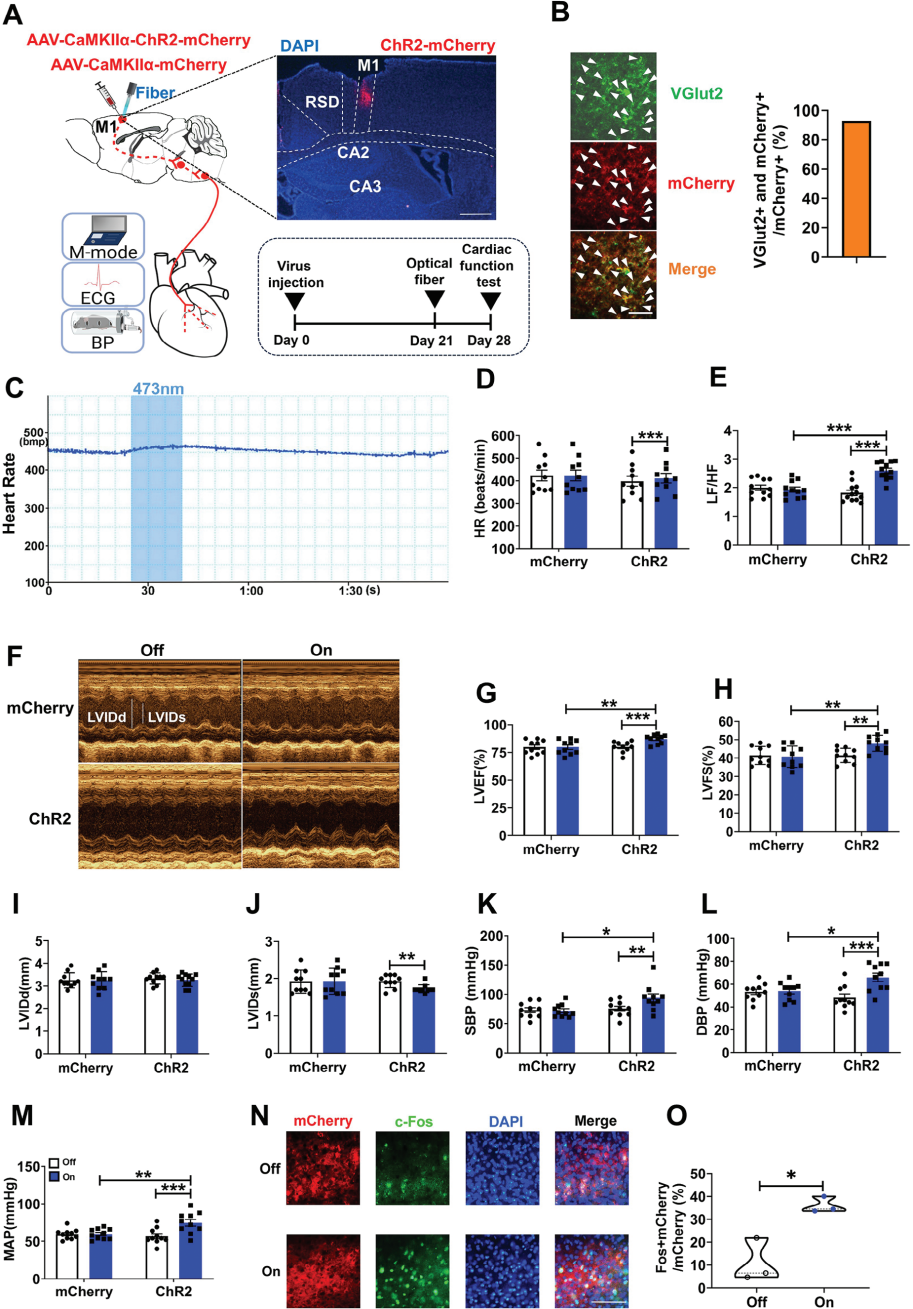
Activating M1 glutamatergic neurons affected heart function and blood pressure. A) Schematic drawing showing viral injection strategy (left), a representative image showing ChR2‐mCherry expression (right top) and the experimental timeline (right bottom), respectively. Scale bar, 500 µm. B) Images showing the overlap between ChR2‐mCherry (red) and vesicular glutamate transporter 2 (VGlut2) (green) in the M1 (left), and the percentage of ChR2 cells expressing VGlut2 (right, n = 4). Scale bar, 50 µm. C) Representative curve graph of HR over time. D, E) Quantification of HR (Two‐way repeated‐measures ANOVA‐Interaction: F (1, 18) = 25.560, *P* < 0.001; mCherry off versus on, *P* = 1.000; ChR2 off versus on, *P* < 0.001; off mCherry versus ChR2, *P* = 0.444; on mCherry versus ChR2, *P* = 0.707; mCherry = 10, ChR2 = 10), LF/HF power ratio (Two‐way repeated‐measures ANOVA‐Interaction: F (1, 21) = 48.314, *P* < 0.001; mCherry off versus on, *P* = 0.406; ChR2 off versus on, *P* < 0.001; off mCherry versus ChR2, *P* = 0.201; on mCherry versus ChR2, *P* < 0.001;mCherry = 11, ChR2 = 12) in anesthetized mice. F) Examples of echocardiographic images in anesthetized mice. G, H, I, J, K, L, M) Quantification of LVEF (Two‐way repeated‐measures ANOVA‐Interaction: F (1, 18) = 68.023, *P* < 0.001; mCherry off versus on, *P* = 0.980; ChR2 off versus on, *P* < 0.001; off mCherry versus ChR2, *P* = 0.933; on mCherry versus ChR2, *P* < 0.01; mCherry = 10, ChR2 = 10), LVFS (Two‐way repeated‐measures ANOVA‐Interaction: F (1, 18) = 16.221, *P* < 0.01; mCherry off versus on, *P* = 0.477; ChR2 off versus on, *P* < 0.01; off mCherry versus ChR2, *P* = 0.984; on mCherry versus ChR2, *P* < 0.01; mCherry = 10, ChR2 = 10), LVIDd (Two‐way repeated‐measures ANOVA‐Interaction: F (1, 18) = 0.137, *P* = 0.715; mCherry off versus on, *P* = 0.897; ChR2 off versus on, *P* = 0.699; off mCherry versus ChR2, *P* = 0.547; on mCherry versus ChR2, *P* = 0.682; mCherry = 10, ChR2 = 10), LVIDs (Two‐way repeated‐measures ANOVA‐Interaction: F (1, 18) = 9.309, *P* < 0.01; mCherry off versus on, *P* = 0.823; ChR2 off versus on, *P* < 0.001; off mCherry versus ChR2, *P* = 0.929; on mCherry versus ChR2, *P* = 0.101; mCherry = 10, ChR2 = 10), SBP (Two‐way repeated‐measures ANOVA‐Interaction: F (1, 18) = 6.232, *P* < 0.05; mCherry off versus on, *P* = 0.830; ChR2 off versus on, *P* < 0.01; off mCherry versus ChR2, *P* = 0.699; on mCherry versus ChR2, *P* < 0.05; mCherry = 10, ChR2 = 10), DBP (Two‐way repeated‐measures ANOVA‐Interaction: F (1, 18) = 22.638, *P* < 0.001; mCherry off versus on, *P* = 0.816; ChR2 off versus on, *P* < 0.001; off mCherry versus ChR2, *P* = 0.699; on mCherry versus ChR2, *P* < 0.05; mCherry = 10, ChR2 = 10) and MAP (Two‐way repeated‐measures ANOVA‐Interaction: F (1, 18) = 20.249, *P* < 0.001; mCherry off versus on, *P* = 0.943; ChR2 off versus on, *P* < 0.001; off mCherry versus ChR2, *P* = 0.521; on mCherry versus ChR2, *P* < 0.01; mCherry = 10, ChR2 = 10) in anesthetized mice. N) Immunohistochemical images showing overlap of ChR2‐mCherry (red) and c‐Fos (green)‐positive neurons in the M1. Scale bar, 100 µm. O) Quantification of c‐Fos‐positive cells in the M1 (Independent t test. t (4) = −4.311, *P*< 0.05). Data are mean ± SEM. *P < 0.05; **P < 0.01, ***P < 0.001.

To test whether M1 glutamatergic neurons affect heart function, we manipulated M1 glutamatergic neurons by optogenetic activation, chemogenetic inactivation and ablation of the M1 excitatory neurons. At first, AAV‐expressing ChR2 driven by the CaMKIIα promoter, which has been shown to be expressed by excitatory glutaminergic neurons,^[^ ^13^ ^]^ was injected into the M1 of adult male mice to activate M1 glutamatergic neurons (**Figure** **2**A). Results confirmed that 92.7% of the ChR2 neurons were glutamatergic (Figure 2B). Light activation significantly increased heart rate (HR) and the low frequency/high frequency (LF/HF) power ratio, which is generally accepted as an index of sympathovagal balance (Figure 2C,D,E). Images of echocardiography were obtained under light‐off or light‐on (Figure 2F). Optogenetic activation of M1 glutamatergic neurons increased left ventricular ejection fraction (LVEF) (Figure 2G), left ventricular fractional shortening (LVFS) (Figure 2H), and decreased left ventricular internal diameter at end‐systole (LVIDs) in mice (Figure 2J). Furthermore, light activation of M1 L5 neurons regulated blood pressure by increasing systolic blood pressure(SBP) (Figure 2K), diastolic blood pressure (DBP) (Figure 2L) and mean artery pressure (MAP) (Figure 2M). There is no difference in left ventricular internal diameter at end‐diastole (LVIDd) (Figure 2I). Meanwhile, optogenetic stimulation of M1 glutamatergic neurons significantly increased the percentage of mCherry and c‐Fos double‐positive neurons compared to no light stimulation of ChR2 mice (Figure 2N,O). When we activated glutamatergic neurons in M2 and S1, no changes were observed in HR, cardiac function, and blood pressure (Figure S2, Supporting Information). These results indicate that activation of the M1 glutamatergic neurons significantly influenced HR, cardiac function, electrical conduction, and blood pressure.

**Figure 3:**
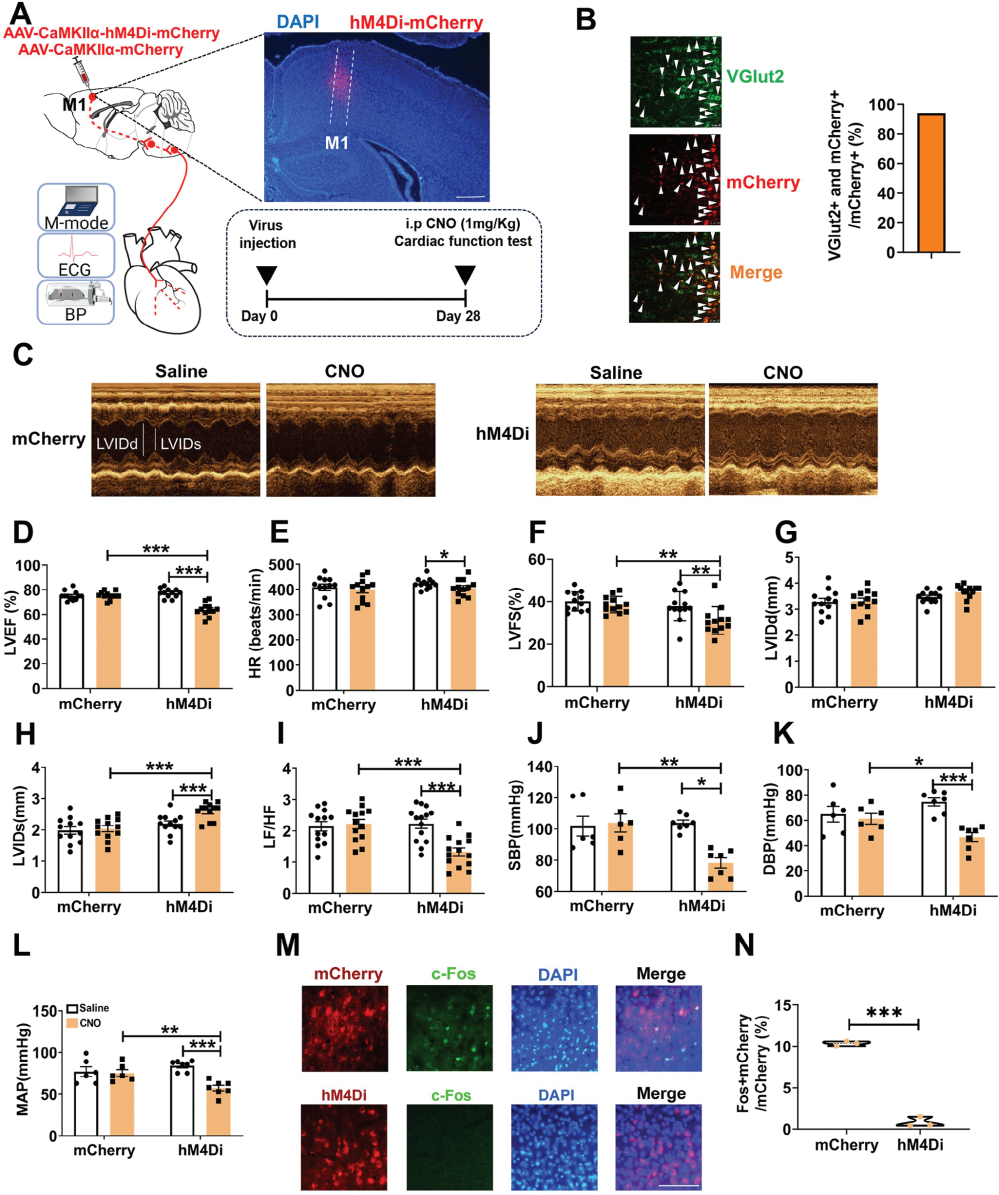
Chemogenetic inhibition of M1 glutamatergic neurons suppressed heart function and blood pressure. A) Experimental schematics (left), a representative image showing hM4Di–mCherry expression in the M1 (right top) and the image of experimental timeline (right bottom), respectively. Scale bar, 500 µm. B) Images showing the overlap between VGlut2 (green) and hM4Di‐mCherry (red) in the M1 (left), and the percentage of hM4Di cells expressing VGlut2 (right, n = 4). Scale bar, 25 µm. C) Examples of echocardiographic images in anesthetized mice. D, E, F, G, H, I) Quantification of LVEF (Two‐way repeated‐measures ANOVA‐Interaction: F (1, 22) = 52.169, *P* < 0.001; mCherry Saline versus CNO, *P* = 0.876; hM4Di Saline versus CNO, *P* < 0.001; Saline mCherry versus hM4Di, *P* = 0.161; CNO mCherry versus hM4Di, *P* < 0.001, mCherry = 12, hM4Di = 12), HR (Two‐way repeated‐measures ANOVA‐Interaction: F (1, 22) = 5.931, *P* < 0.05; mCherry Saline versus CNO, *P* = 0.267; hM4Di Saline versus CNO, *P* < 0.05; Saline mCherry versus hM4Di, *P* = 0.286; CNO mCherry versus hM4Di, *P* = 0.740; mCherry = 12, hM4Di = 12), LVFS (Two‐way repeated‐measures ANOVA‐Interaction: F (1, 22) = 8.603, *P* < 0.01; mCherry Saline versus CNO, *P* = 0.452; hM4Di Saline versus CNO, *P* < 0.01; Saline mCherry versus hM4Di, *P* = 0.341; CNO mCherry versus hM4Di, *P* < 0.01; mCherry = 12, hM4Di = 12), LVIDd (Two‐way repeated‐measures ANOVA‐Interaction: F (1, 22) = 2.420, *P* = 0.134; mCherry Saline versus CNO, *P* = 0.819; hM4Di Saline versus CNO, *P* = 0.062; Saline mCherry versus hM4Di, *P* = 0.245; CNO mCherry versus hM4Di, *P*< 0.05; mCherry = 12, hM4Di = 12), LVIDs (Two‐way repeated‐measures ANOVA‐Interaction: F (1, 22) = 13.221, *P* < 0.01; mCherry Saline versus CNO, *P* = 0.552; hM4Di Saline versus CNO, *P* < 0.001; Saline mCherry versus hM4Di, *P* = 0.155; CNO mCherry versus hM4Di, *P*< 0.001; mCherry = 12, hM4Di = 12) and LF/HF (Two‐way repeated‐measures ANOVA‐Interaction: F (1, 25) = 95.277, *P* < 0.001; mCherry Saline versus CNO, *P* = 0.392; hM4Di Saline versus CNO, *P* < 0.001; Saline mCherry versus hM4Di, *P* = 0.691; CNO mCherry versus hM4Di, *P* < 0.001, mCherry = 13, hM4Di = 14) in anesthetized mice. J, K, L) Quantification of SBP (Two‐way repeated‐measures ANOVA‐Interaction: mCherry Saline versus CNO, *P* = 0.528; hM4Di Saline versus CNO, *P* < 0.05; Wilcoxon rank sum test; Saline mCherry versus hM4Di, *P* = 0.366; CNO mCherry versus hM4Di, *P* < 0.01; two‐sided Mann–Whitney test. mCherry = 6, hM4Di = 7), DBP (Two‐way repeated‐measures ANOVA‐Interaction: F (1, 11) = 7.796, *P* < 0.01; mCherry Saline versus CNO, *P* = 0.578; hM4Di Saline versus CNO, *P* < 0.001; Saline mCherry versus hM4Di, *P* = 0.174; CNO mCherry versus hM4Di, *P* < 0.05, mCherry = 6, hM4Di = 7) and MAP (Two‐way repeated‐measures ANOVA‐Interaction: F (1, 11) = 11.536, *P* < 0.01; mCherry Saline versus CNO, *P* = 0.764; hM4Di Saline versus CNO, *P* < 0.001; Saline mCherry versus hM4Di, *P* = 0.261; CNO mCherry versus hM4Di, *P* < 0.01, mCherry = 6, hM4Di = 7) in conscious mice. M) Immunohistochemical images showing overlap of hM4Di (red) and c‐Fos (green) neurons in the M1 expressing mCherry or hM4Di mice. Scale bar, 100 µm. N) Quantification of c‐Fos‐positive cells in the M1 (Independent t test. t (4) = 24.356, *P* < 0.001). Data are mean ± SEM. *P < 0.05; **P < 0.01, ***P < 0.001.

Next, we injected AAV expressing hM4Di by the CaMKIIα promoter into the M1 (**Figure** **3**A). Among hM4Di neurons, 94.3% were glutamatergic (Figure 3B). After four weeks, images of echocardiography were obtained after saline or clozapine‐N‐oxide (CNO) injection (Figure 3C). Chemogenetic inactivation of M1 glutamatergic neurons decreased LVEF (Figure 3D), HR (Figure 3E), LVFS (Figure 3F), LF/HF power ratio (Figure 3I), SBP (Figure 3J), DBP (Figure 3K), and MAP (Figure 3L), but increased LVIDs (Figure 3H). No significant changes were observed in LVIDd (Figure 3G). After CNO administration, the percentage of mCherry and c‐Fos overlapping neurons was significantly lower in hM4Di mice compared to control mice, indicating that chemogenetic suppression of M1 glutamatergic neurons (Figure 3M,N). These results suggest that chemogenetically inhibiting the M1 neurons significantly reduced HR, heart function, and blood pressure.

**Figure 4:**
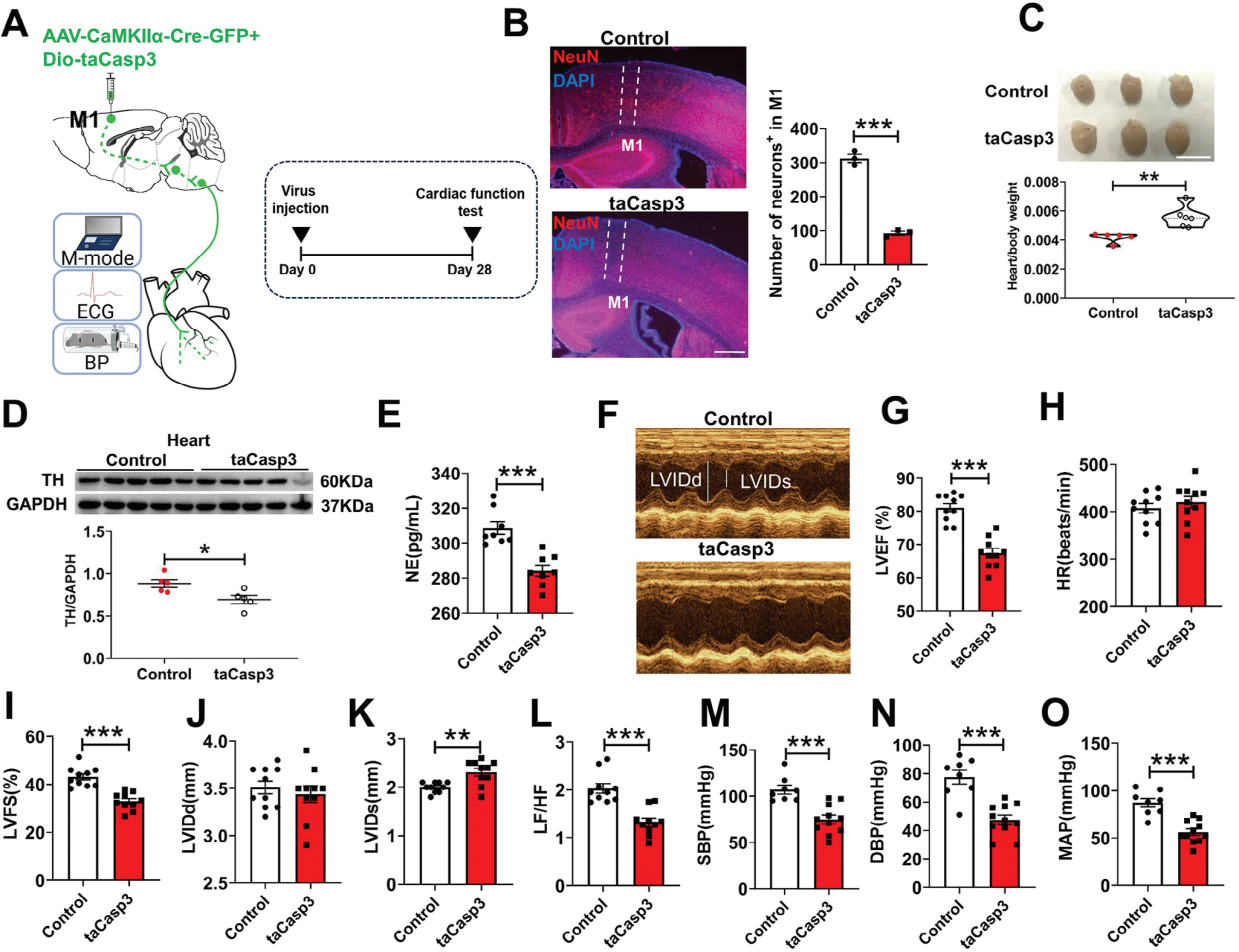
M1 glutamatergic neuronal ablation impaired heart function and reduced blood pressure. A) Experimental schematics (left) and timeline (right). B) Representative images showing NeuN‐positive neurons (red) in M1(left) and the number of neurons^+^ in M1 (t (4) = 6.884, *P* < 0.01 by independent t‐tests; Control = 3, taCasp3 = 3) (right), respectively. Scale bar, 500 µm. C) Ablating M1 glutamatergic neurons increased the ratio of heart/body weight (t (9) = −3.921, *P* < 0.01 by independent t‐tests; Control = 5, taCasp3 = 6). D) Effect of ablating M1 glutamatergic neurons on TH protein levels of left ventricular tissues (t (8) = 2.787, *P* < 0.05 by independent t‐tests; Control = 5, taCasp3 = 5). E) The NE content of left ventricular myocardium (t (14) = 5.013, *P*<0.001 by independent t‐tests; Control = 8, taCasp3 = 8). F) Examples of echocardiographic images in anesthetized mice. G, H, I, J, K, L) Quantification of LVEF (t (18) = 7.131, *P*<0.001 by independent t‐tests; Control = 10, taCasp3 = 10), HR (t (18) = −0.182, *P* = 0.428 by independent t‐tests; Control = 10, taCasp3 = 10), LVFS (t (18) = 27.854, *P*<0.001 by independent t‐tests; Control = 10, taCasp3 = 10), LVIDd (t (18) = 0.629, *P* = 0.537 by independent t‐tests; Control = 10, taCasp3 = 10); LVIDs (t(18) = −3.543, *P*<0.01 by independent t‐tests; Control = 10, taCasp3 = 10) and LF/HF radio(t (18) = 6.416, *P* < 0.001 by independent t‐tests; Control = 10, taCasp3 = 10) in anesthetized mice. M, N, O) Quantification of SBP (t (17) = 4.662, *P* < 0.001 by independent t‐tests; Control = 8, taCasp3 = 11), DBP (t (17) = 5.281, *P*< 0.001 by independent t‐tests; Control = 8, taCasp3 = 11) and MAP (t (17) = 5.476, *P*< 0.001 by independent t‐tests; Control = 8, taCasp3 = 11) in conscious mice. Data are mean ± SEM.*P < 0.05; **P < 0.01, ***P < 0.001.

We also ablated the M1 excitatory neurons by co‐injecting AAV‐EF1α‐DIO‐taCasp3‐TEVp and AAV‐CaMKIIα‐Cre‐GFP, which triggered cell apoptosis by inducing the expression of genetic engineering Caspase‐3 (taCasp3) (**Figure** **4**A).^[^ ^14^ ^]^ M1 glutamatergic neuronal ablation was confirmed by loss of NeuN (a mature neuronal marker)‐positive neurons in taCasp3 mice (Figure 4B). The heart weight/body weight (HW/BW) was much smaller in control mice compared with taCasp3 mice (Figure 4C). Ablation of M1 glutamatergic neurons also reduced tyrosine hydroxylase (TH) protein expression and norepinephrine (NE) levels in the left ventricle of mice (Figure 4D,E). Representative images of echocardiography were obtained in control and taCasp3 mice (Figure 4F). Compared with control mice, M1 glutamatergic neurons ablation showed a significant reduction in LVEF (Figure 4G), LVFS (Figure 4I), LF/HF power ratio（Figure 4L）, SBP (Figure 4M), DBP (Figure 4N), and MAP (Figure 4O), and an increase in LVIDs (Figure 4K). However, the HR and LVIDd don't show any difference (Figure 4H,J).

### 2.3 The M1 to MnR Pathway is Crucial in Regulating Cardiac Function and Blood Pressure in Normal Mice

**Figure 5:**
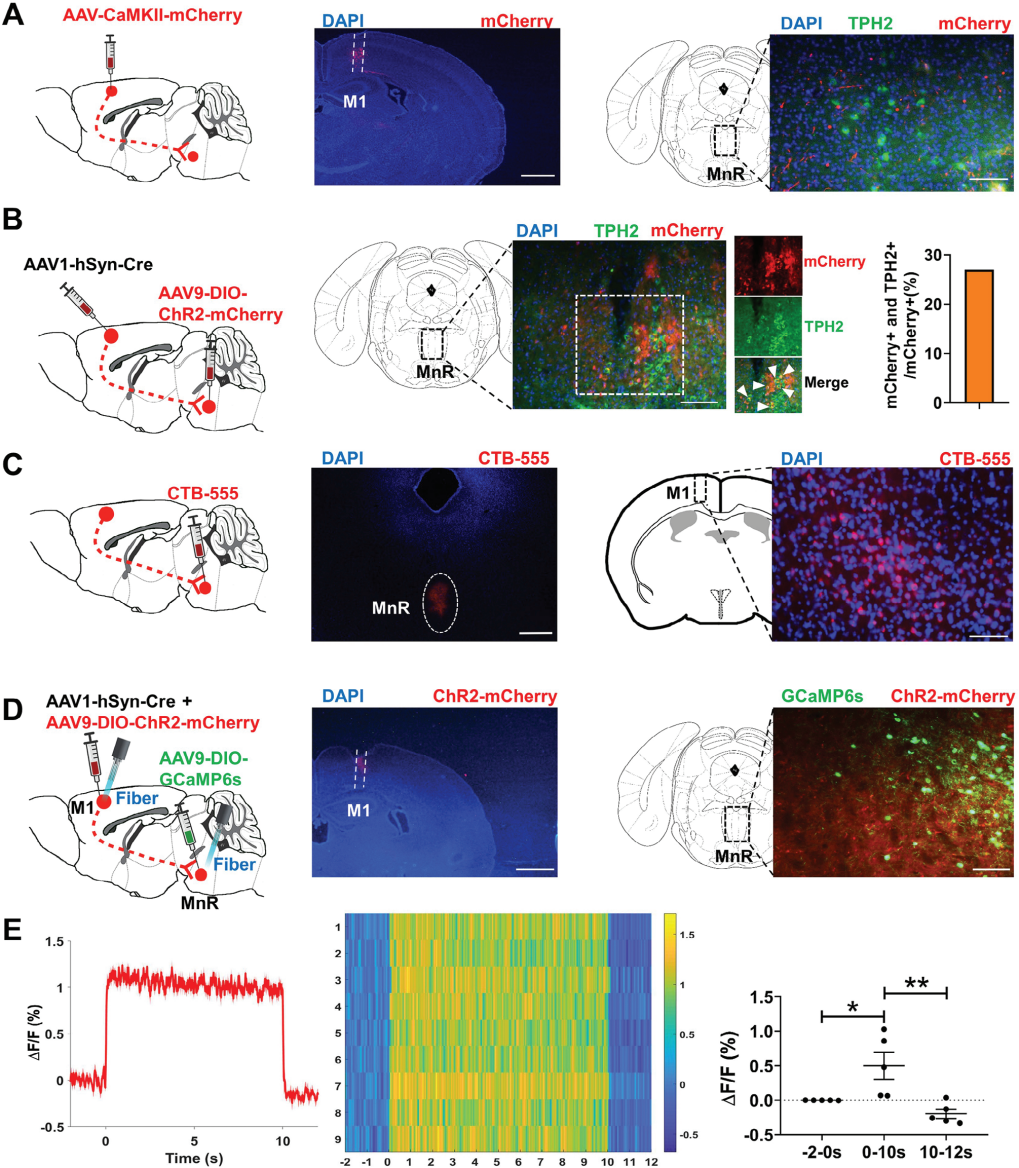
M1 neurons input to MnR. A) Experimental design (left), representative images of mCherry‐labeled M1 neurons (middle) and their fibers in the MnR (right), respectively. Scale bar, 500 µm (middle), 100 µm (right). B) Schematic illustration of hSyn‐CRE virus injection in the M1 and DIO‐ChR2‐mCherry virus injection in the MnR (left), images showing the overlap between ChR2‐mCherry (red) and TPH2 (green) in the M1(middle), and the ratio of TPH2‐positive neurons (right), respectively. Scale bar, 200 µm. C) Experimental design (left), and retrogradely labeled neurons in the M1 (right) by injection of CTB555 into the MnR (middle). Scale bar, 500 µm (middle); 200 µm (right). D) Strategy of viral injection, optogenetic and photometric recording (left); representative images of ChR2‐mCherry‐labeled M1 neurons (middle) and representative images of GCaMP6s^+^ neurons and ChR2^+^ fibers in MnR (right), respectively. Scale bar, 500 µm (middle), 100 µm (right). E) A representative trace of calcium signals recorded in MnR (left), heatmap showing fluorescence signals (middle) and quantification of calcium signals (right) (One‐way ANOVA. baseline versus signal, *P* < 0.05; signal versus poststimulation, *P* < 0.01; baseline versus poststimulation, *P* = 0. 508; n = 5), respectively. Data are mean ± SEM. *P < 0.05; **P < 0.01.

To investigate the M1 projections and synaptic targets, we infected M1 neurons with AAV‐CaMKIIα‐EGFP. The brain regions most intensely labeled by EGFP were the medial prefrontal cortex (mPFC), the parafascicular thalamic nucleus (PF), the basolateral amygdaloid nucleus, the MnR, the locus coeruleus (LC) and the pyramidal tract, which are involved in the control of heart function (Figure S3A, B, Supporting Information). Trans‐synaptic anterograde tracing using vesicular stomatitis virus encoding EGFP into the M1 revealed fluorescently labeled neurons in the same brain areas (Figure S3C, D, Supporting Information). Tracing axons from mCherry‐labeled M1 glutamatergic neurons revealed strong projection to the MnR (**Figure** **5**A). Immunostaining showed that tryptophan hydroxylase 2 (TPH2)‐positive neurons in MnR were in proximity from M1 glutamatergic fibers (Figure 5A). When AAV1‐hSyn‐Cre was injected into the M1 and DIO‐ChR2‐mCherry was injected into the MnR, we found that 25% of neurons projecting from M1 to MnR expressed TPH2 (Figure 5B). Injection of CTB555 into the MnR resulted in retrograde labeling of the cells in the M1 (Figure 5C). To record the activities of the MnR neurons, anterograde transneuronal transport of AAV1‐hSyn‐Cre and AAV9‐DIO‐ChR2‐mCherry were injected into the M1, and then AAV9‐DIO‐GCaMP6s was delivered into the MnR, and fiber photometry was performed (Figure 5D). We found a significant increase in the intensity of fluorescent signals of the MnR when experimental mice received blue light stimulation (Figure 5E). Taken together, the results revealed that MnR neurons receive direct input from the M1.

**Figure 6:**
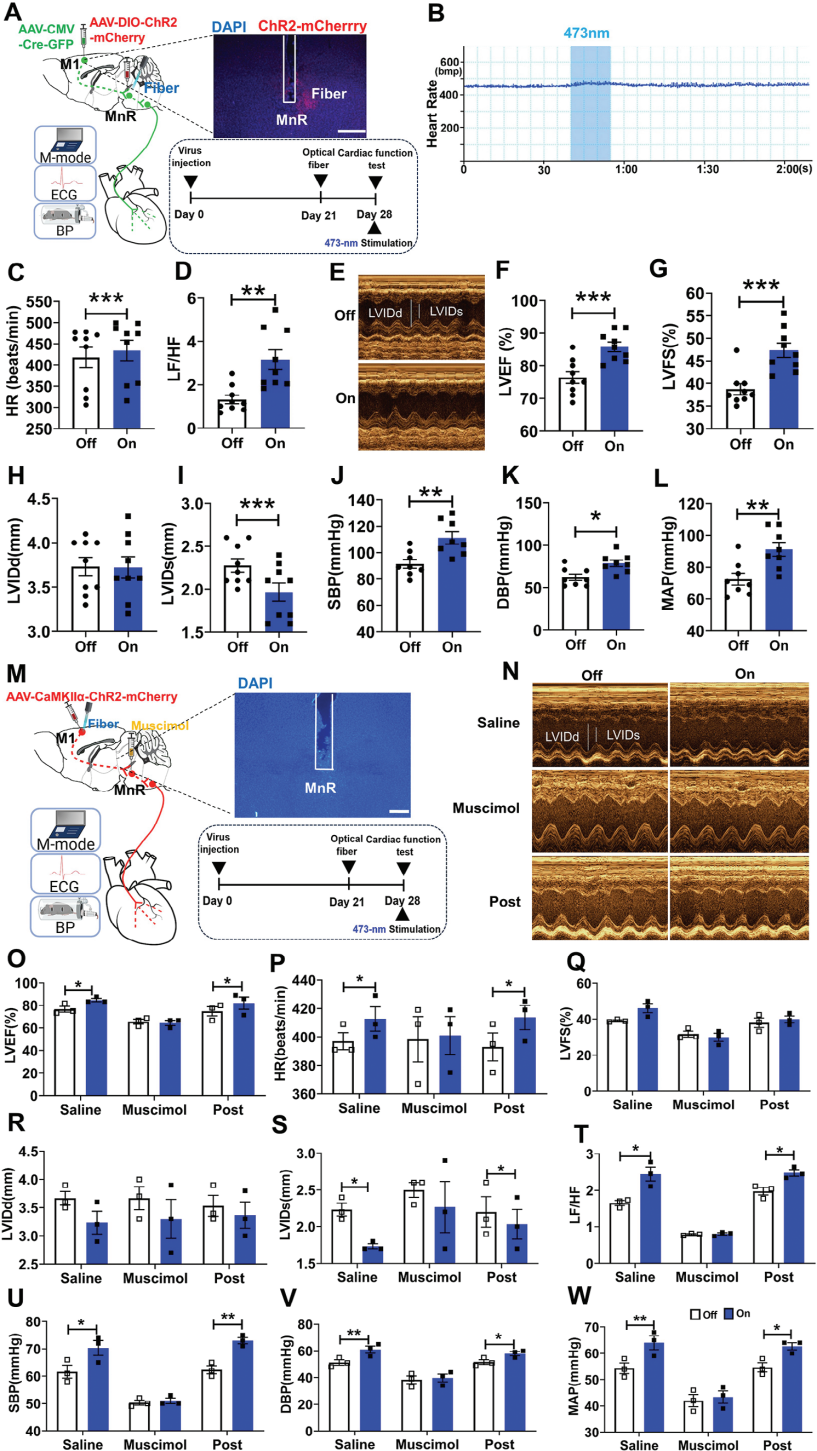
Optogenetic activation of MnR‐projecting neurons of the M1 affected cardiac function. A) Experimental designs (left panel), a representative image showing ChR2‐mCherry expression in MnR and optic fiber implantation above the MnR (right top), and the experimental timeline of the image (right bottom). Scale bar, 500 µm. B) Representative curve graph of heart rate over time. C, D) Quantification of HR (t (8) = −5.109, *P* < 0.001 by paired t test; n = 9) and LF/HF (t (8) = −4.032, *P* < 0.01 by paired t test; n = 9). E) Examples of echocardiographic images in anesthetized mice. F, G, H, I, J, K, L) Quantification of LVEF (t (8) = −11.854, *P* < 0.001 by paired t test; n = 9), LVFS (t(8) = −8.042, *P* < 0.001 by paired t test; n = 9), LVIDd (t (8) = 0.164, *P* = 0.873 by paired t test; n = 9), LVIDs (t (8) = 6.424, *P* < 0.001 by paired t test; n = 9), SBP (t (7) = −4.494, *P* < 0.01 by paired t test; n = 8), DBP (t (7) = −2.855, *P* < 0.05 by paired t test; n = 8) and MAP (t(7) = −4.131, *P* < 0.01 by paired t test; n = 8) in anesthetized mice. M) Schematic of combined optogenetic and pharmacology (left), a representative image showing the location of injector and fiber tip in the MnR (right top) and the image of experimental timeline (right bottom). Scale bar, 1 mm. N) Examples of echocardiographic images in anesthetized mice. O, P, Q, R, S, T, U, V, W) Quantification of LVEF (Saline: t (2) = −6.363, *P*< 0.05 by paired t test; muscimol: t (2) = 1.936, *P* = 0.192 by paired t test; Post: t (2) = −5.765, *P* < 0.05 by paired t test; n = 3), HR (Saline: t (2) = −4.631, *P* < 0.05 by paired t test; muscimol: t (2) = −1, *P* = 0.423 by paired t test; Post: t (2) = −8.598, *P* < 0.05 by paired t test; n = 3), LVFS (Saline: t (2) = −2.666, *P* = 0.117 by paired t test; muscimol: t (2) = 0.736, *P* = 0.0.539 by paired t test; Post: Saline: t (2) = −2.238, *P* = 0.155 by paired t test; n = 3), LVIDd (Saline: t (2) = 3.250, *P* = 0.083 by paired t test; muscimol: t (2) = 0.780, *P* = 0.517 by paired t test; Post: t (2) = 2.5, *P* = 0.130 by paired t test; n = 3), LVIDs (Saline: t (2) = 0.660, *P* < 0.05 by paired t test; muscimol: t (2) = 0.661, *P* = 0.576 by paired t test; Post: t (2) = 5, *P* < 0.05 by paired t test; n = 3) and LF/HF (Saline: t (2) = −4.289, *P* < 0.05 by paired t test; muscimol: t (2) = −0.301, *P* = 0.792 by paired t test; Post: t (2) = −6.830, *P* < 0.05 by paired t test; n = 3), SBP (Saline: t (2) = −7.221, *P* < 0.05 by paired t test; muscimol: t (2) = −2, *P* = 0.184 by paired t test; Post: t (2) = −31, *P* < 0.01 by paired t test; n = 3), DBP (Saline: t (2) = −10.961, *P* < 0.01 by paired t test; muscimol: t (2) = −4, *P* = 0.057 by paired t test; Post: t (2) = −4.359, *P* < 0.05 by paired t test; n = 3), MAP (Saline: t (2) = −10.961, *P* < 0.05 by paired t test; muscimol: t (2) = −4, *P* = 0.057 by paired t test; Post: t (2) = −6.982, *P* < 0.05 by paired t test; n = 3) in anesthetized mice before, during, and after muscimol infusion into the MnR. Data are mean ± SEM. *P < 0.05; **P < 0.01, ***P < 0.001.

Furthermore, we hypothesized that the M1‐MnR pathway was required for regulating cardiac function and blood pressure. To test this hypothesis, we injected anterograde transneuronal transport of AAV1‐CMV‐Cre‐GFP into the M1 and AAV9‐DIO‐ChR2‐mCherry into the MnR (**Figure** **6**A). We specifically activated ChR2 in MnR neurons postsynaptic to the M1 by implanting optical fibers above the MnR, and then tested the cardiac function (Figure 6A). Optogenetic activation of M1‐MnR neuron projections increased HR and the LF/HF power ratio (Figure 6B,C,D). Representative images of echocardiography were obtained in light‐on and light‐off, respectively (Figure 6E). We observed an increase in LVEF and LVFS, and a decrease in LVIDs (Figure 6F,G,I), and no difference in LVIDd (Figure 6H). Blood pressure detection showed that SBP, DBP, and MAP significantly increased after activation of M1 L5 neurons (Figure 6J,K,L).

Meanwhile, we also expressed ChR2 in the M1 neurons and optical fibers were implanted above the M1 (Figure 6M). Similarly, the result showed that photostimulation of M1 to MnR projections were sufficient to influence heart function, blood pressure and the balance of autonomic neural activities (Figure 6N–W). The effect was prevented when muscimol was infused into the MnR (Figure 6N–W), suggesting that MnR neuronal activity is necessary for this effect.

### 2.4 Manipulation of M1 Glutamatergic Neurons Affected Cardiac Function and Blood Pressure in Mice with MI

**Figure 7:**
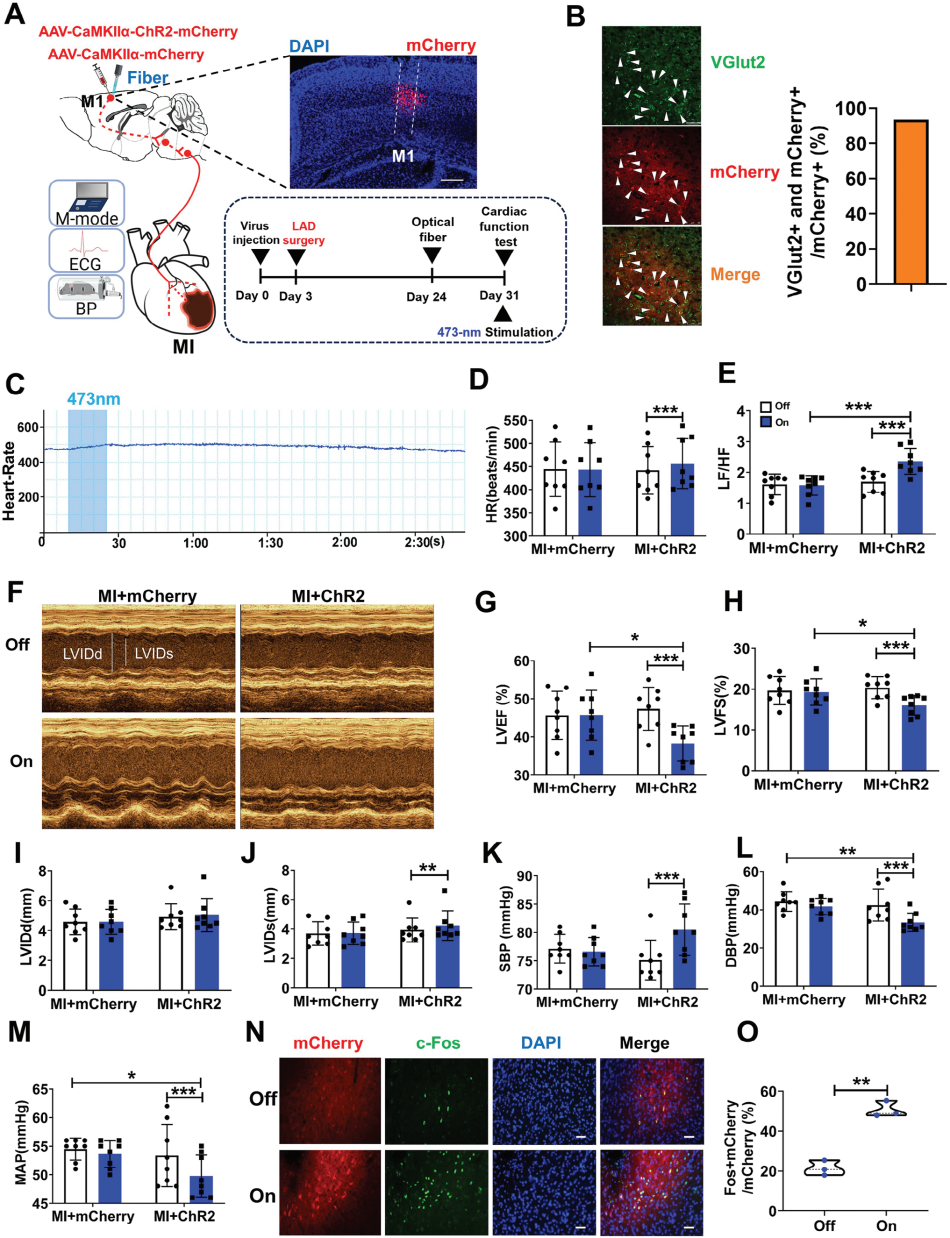
Optogenetic activating the M1 excitatory neurons weakened heart function in MI mice. A) Experimental operation diagram (left), a representative image showing ChR2–mCherry expression (right top), and image of experimental timeline (right bottom). Scale bar, 500 µm. B) Colocalization of ChR2‐mCherry expression (red) and VGlut2 staining (green) (left) and the percentage of ChR2 cells expressing VGlut2 (right, n = 4). Scale bar, 50 µm. C) Representative curve graph of HR over time. D, E) Quantification of HR (Two‐way repeated‐measures ANOVA‐Interaction: F (1, 14) = 11.503, *P*< 0.01; mCherry off versus on, P = 0.693; ChR2 off versus on, *P*< 0.001; off mCherry versus ChR2, P = 0.929; on mCherry versus ChR2, P = 0.649; mCherry = 8, ChR2 = 8), LF/HF (Two‐way repeated‐measures ANOVA‐Interaction: F (1, 14) = 30.469, *P*<0.05, mCherry off versus on, *P* = 0.685; ChR2 off versus on, *P* < 0.001; off mCherry versus ChR2, *P* = 0.621; on mCherry versus ChR2, *P* < 0.001; mCherry = 8, ChR2 = 8). F) Examples of echocardiographic images in anesthetized mice. G, H, I, J, K, L, M,) Quantification of LVEF (Two‐way repeated‐measures ANOVA‐Interaction: F (1, 14) = 71.675, *P* < 0.01; mCherry off versus on, *P* = 0.943; ChR2 off versus on, *P* < 0.001; off mCherry versus ChR2, *P* = 0.576; on mCherry versus ChR2, *P* < 0.05; mCherry = 8, ChR2 = 8), LVFS (Two‐way repeated‐measures ANOVA‐Interaction: F (1, 14) = 37.954, *P* < 0.001; mCherry off versus on, *P* = 0.496; ChR2 off versus on, *P* < 0.001; off mCherry versus ChR2, *P* = 0.664; on mCherry versus ChR2, *P* < 0.05; mCherry = 8, ChR2 = 8), LVIDd (Two‐way repeated‐measures ANOVA‐Interaction: F (1, 14) = 0.533; mCherry off versus on, *P* = 1; ChR2 off versus on, *P* = 0.319; off mCherry versus ChR2, *P* = 0.447; on mCherry versus ChR2, *P* = 0.376; mCherry = 8, ChR2 = 8), LVIDs (Two‐way repeated‐measures ANOVA‐Interaction: F (1, 14) = 5.316, *P* < 0.05; mCherry off versus on, *P* = 0.898; ChR2 off versus on, *P* < 0.01; off mCherry versus ChR2, *P* = 0.565; on mCherry versus ChR2, *P* = 0.261; mCherry = 8, ChR2 = 8), SBP (Two‐way repeated‐measures ANOVA‐Interaction: F (1, 14) = 15.154, *P* < 0.01; mCherry off versus on, *P* = 0.239; ChR2 off versus on, *P* < 0.001; off mCherry versus ChR2, *P* = 0.052; on mCherry versus ChR2, *P* = 0.213; mCherry = 8, ChR2 = 8), DBP (Two‐way repeated‐measures ANOVA‐Interaction: F (1, 14) = 39.035, *P* < 0.01; mCherry off versus on, *P* = 0.581; ChR2 off versus on, *P* < 0.001; off mCherry versus ChR2, *P* = 0.595; on mCherry versus ChR2, *P*<0.01; mCherry = 8, ChR2 = 8), MAP (Two‐way repeated‐measures ANOVA‐Interaction: F (1, 14) = 19.982, *P* < 0.01; mCherry off versus on, *P* = 0.066; ChR2 off versus on, *P* < 0.001; off mCherry versus ChR2, *P* = 0.591; on mCherry versus ChR2, *P*<0.05; mCherry = 8, ChR2 = 8) in anesthetized mice. N). Immunohistochemical images showing overlap of ChR2‐mCherry (red) and c‐Fos (green)‐positive neurons in the M1. Scale bar, 100 µm. O) Quantification of c‐Fos‐positive cells in the M1 (Independent t test. t (4) = −9.282, *P*< 0.01). Data are mean ± SEM. *P < 0.05; **P < 0.01, ***P < 0.001.

To test whether modulation of M1 glutamatergic neuronal activity influenced cardiac function under the pathological conditions, we virally expressed mCherry‐tagged ChR2 in the M1 glutamatergic neurons and implanted optical fibers bilaterally in MI mice (**Figure** **7**A). The results showed that 93.6% of neurons labeled with ChR2 were glutamatergic neurons (Figure 7B) and that light activation significantly increased HR and the LF/HF power ratio (Figure 7C,D,E) and aggravated cardiac function by increasing LVIDs (Figure 7J) and reducing LVEF and LVFS (Figure 7G,H). It's no different at LVIDd (Figure 7I). Meanwhile, it also increased SBP but decreased DBP and MAP (Figure 7K,L,M). In addition, the proportion of mCherry and c‐Fos double‐positive neurons increased significantly under light stimulation in the MI+ChR2 group (Figure 7N,O). These results show that optogenetic activation of M1 glutamatergic neurons had an adverse effect on cardiac function in MI mice.

Next, we injected the AAV‐expressing hM4Di driven by CaMKIIα promoter into M1 region of the mice with MI, then ligation of the left anterior descending coronary artery and stimulated with CNO injection once a day for 21 consecutive days (Figure S4A, B, Supporting Information). Post hoc histology confirmed that the expression of hM4Di‐mCherry was largely restricted to L5 neurons in the M1 region (Figure S4A, Supporting Information). Echocardiography of MI+mCherry group and MI+ hM4Di group mice were obtained after continuous intraperitoneal injection of CNO for 21 days (Figure S4C, Supporting Information). We found that inhibition of M1 glutamatergic neurons after MI significantly increased LVEF, and LVFS, but decreased HR, LVIDd, LVIDs and the LF/FH power ratio (Figure S4D–I, Supporting Information). Meanwhile, the DBP and MAP were increased in MI+ hM4Di group mice (Figure S4K,L, Supporting Information), but SBP did not change (Figure S4J, Supporting Information). CNO injection significantly inhibited the activity of M1 glutamatergic neurons by reducing c‐Fos expression (Figure S4M,N, Supporting Information). These results demonstrate that the M1 glutamatergic neurons significantly affected heart function and blood pressure in MI mice.

**Figure 8:**
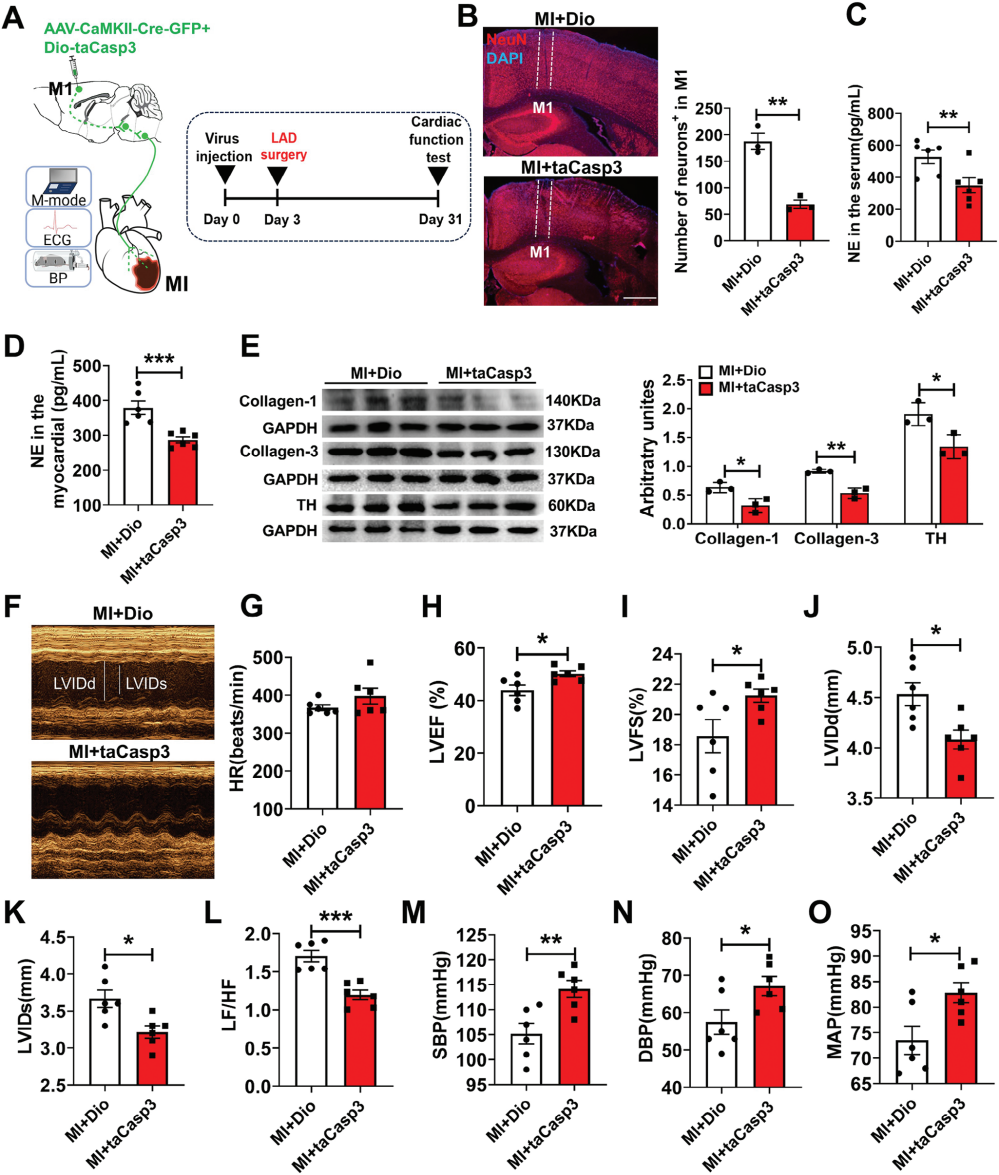
Ablating M1 excitatory neurons improved cardiac function in MI mice. A) Experimental schematics and timeline. B) Image showing a NeuN signals (red) in M1. Scale bar, 500 µm (left), and the number of neurons in M1 (right panel; t (4) = 16.102, *P*< 0.001 by independent t‐tests; MI+Dio = 3, MI+taCasp3 = 3). Scale bar = 200 µm. C) The content of serum NE t (10) = 2.812, *P* < 0.01 by independent t‐tests; MI+Dio = 6, MI+taCasp3 = 6. D) The NE content of left ventricular myocardium (t (10) = 4.381, *P* < 0.01 by independent t‐tests; MI+Dio = 6, MI+taCasp3 = 6). E) Expression of cardiac protein in the marginal zone of left ventricular infarction (Collagen‐1 protein levels of heart: t (4) = 3.421, *P*< 0.05 by independent t‐tests; MI+Dio = 3, MI+taCasp3 = 3. Collagen‐3 protein levels of heart: t (4) = 7.021, *P*< 0.01 by independent t‐tests; MI+Dio = 3, MI+taCasp3 = 3. TH protein levels of heart: t (4) = 3.403, *P*< 0.05 by independent t‐tests; MI+Dio = 3, MI+taCasp3 = 3). F) Examples of echocardiographic images in anesthetized mice. G, H, I, J, K, L) Quantification of HR (t (12) = −1.385, P = 0.196 by independent t‐tests; MI+Dio = 6, MI+taCasp3 = 6), LVEF (t(10) = −2.737, *P*< 0.05 by independent t‐tests; MI+Dio = 6, MI+taCasp3 = 6), LVFS (t(10) = −2.264, *P*< 0.05 by independent t‐tests; MI+Dio = 6, MI+taCasp3 = 6), LVIDd (t(10) = 3.03, *P*<0.05 by independent t‐tests; MI+Dio = 6, MI+taCasp3 = 6), LVIDs (t(10) = 3.126, *P*< 0.05 by independent t‐tests; MI+Dio = 6, MI+taCasp3 = 6), LF/HF (t (10) = 5.116, *P*< 0.001 by independent t‐tests; MI+Dio = 6, MI+taCasp3 = 6) in anesthetized mice. M, N, O) SBP (t (10) = −3.372, *P*< 0.01 by independent t‐tests; MI+Dio = 6, MI+taCasp3 = 6), DBP (t (10) = −2.330, *P*< 0.05 by independent t‐tests; MI+Dio = 6, MI+taCasp3 = 6) and MAP (t (10) = −2.731, *P*< 0.05 by independent t‐tests; MI+Dio = 6, MI+taCasp3 = 6) in conscious mice. Data are mean ± SEM. *P < 0.05; **P < 0.01, ***P < 0.001.

We also ablated M1 excitatory neurons by co‐injection of AAV‐CaMKIIα‐Cre‐EGFP and AAV‐CAG‐DIO‐taCasp3‐TEVp in M1 neurons of the MI mice (**Figure** **8**A). Postmortem immunostaining showed the expression of CaMKIIα‐Cre‐EGFP in M1 L5 region, and resulted in a significant decrease in NeuN cell density at the injection site (Figure 8B). NE in serum and left ventricular myocardium were significantly reduced in MI+taCasp3‐ablated mice compared with the control group (Figure 8C,D); the trend was like cardiac TH expression (Figure 8E). Although Masson staining did not show a significant difference between MI+Dio and MI+taCasp3 (Figure S5A, B, Supporting Information), the expression of collagen‐1 and collagen‐3 proteins were also significantly decreased in left ventricular myocardium (Figure 8E). Moreover, LVEF (Figure 8H), LVFS (Figure 8I), SBP, DBP, and MAP (Figure 8M,N,O) were significantly increased, and LVIDd, LVIDs, and LF/HF power ratio (Figure 8J,K,L) were significantly decreased in taCasp3‐ablated MI mice. Overall, these data suggest that M1 excitatory neurons may be involved in the regulation of cardiac function after MI.

### 2.5 The M1 Neurons that Project to MnR Mediated Cardiac Function in Mice with MI

**Figure 9:**
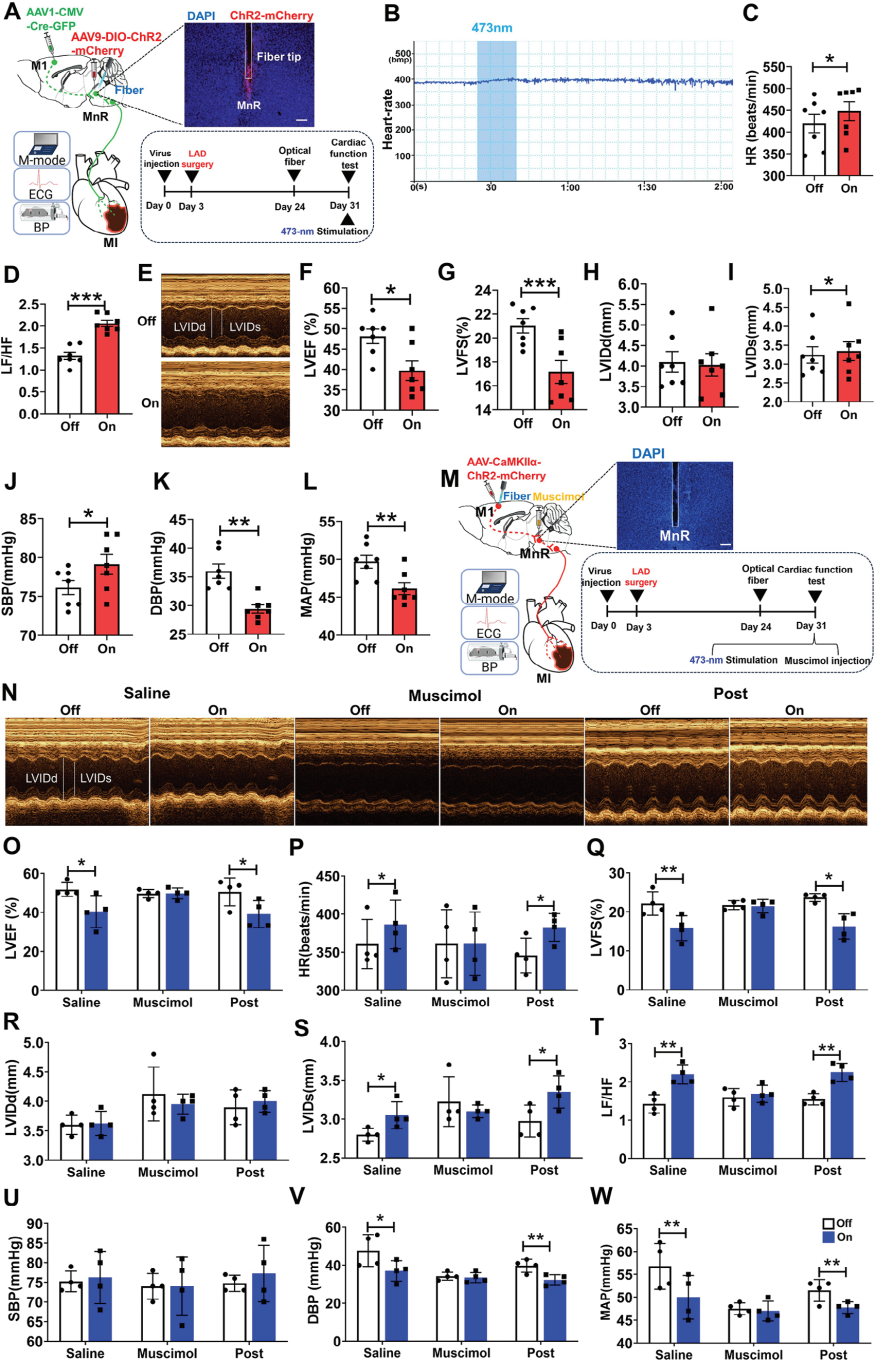
MnR neurons were downstream of the M1 to affect cardiac function in MI mice. A) Experimental schematics (left), a representative image shows ChR2‐mCherry expression in the MnR and optic fiber implantation above the MnR (right top) and image of experimental timeline (right bottom). Scale bar, 100 µm. B) Representative curve graph of HR over time. C, D) Quantification of HR (t (6) = −3.622, *P*< 0.05 by paired t test; n = 7), LF/HF (t (6) = −7.310, *P*< 0.001 by paired t test; n = 7). E) Examples of echocardiographic images in anesthetized mice. F, G, H, I, J, K, L) Quantification of LVEF (t (6) = 3.078, *P*< 0.05 by paired t test; n = 7), LVFS (t (6) = 4.335, *P*< 0.001 by paired t test; n = 7), LVIDd(t (6) = 0.460, *P* = 0.662 by paired t test; n = 7), LVIDs (t (6) = −0.735, *P* = 0.480 by paired t test; n = 7), SBP (t (6) = −2.806, *P*< 0.05 by paired t test; n = 7), DBP (t (6) = 5.499, *P*< 0.01 by paired t test; n = 7), MAP (t (6) = 5.029, *P*< 0.01 by paired t test; n = 7) in anesthetized mice with MI. M) Schematic of experimental and combined optogenetic and pharmacology (left), a representative image showing the location of injector and fiber tip in the MnR (right top), and image of experimental timeline (right bottom). Scale bar, 100 µm. N) Examples of echocardiographic images in anesthetized mice with MI. O, P, Q, R, S, T, V, U, W) Quantification of LVEF (Saline: t (3) = 3.871, *P* < 0.05 by paired t test; muscimol: t (3) = −0.421, *P* = 0.702 by paired t test; Post: t(3) = 3.997, *P*< 0.05 by paired t test; n = 4), HR (Saline: t (3) = −3.402, *P*< 0.05 by paired t test; muscimol: t (3) = 0, *P* = 1 by paired t test; Post: t (3) = −3.623, *P*< 0.05 by paired t test; n = 4), LVFS (Saline: t (3) = 6.249, *P* < 0.01 by paired t test; muscimol: t (3) = 0.419, *P* = 0.703 by paired t test; Post: t (3) = 4.733, *P*< 0.05 by paired t test; n = 4), LVIDd (Saline: t (3) = −0.243, *P* = 0.824 by paired t test; muscimol: t (3) = 0.906, *P* = 0.432 by paired t test; Post: t (2) = −0.562, *P* = 0.613 by paired t test; n = 4); LVIDs (Paired t test. Saline: t (3) = −3.873, *P* < 0.05 by paired t test; muscimol: t (2) = 0.951, *P* = 0.412 by paired t test; Post: t (3) = −3.400, *P*< 0.05 by paired t test; n = 4), LF/HF (Saline: t (3) = −7.996, *P* < 0.01 by paired t test; muscimol: t (3) = −1.580, *P* = 0.212 by paired t test; Post: t (3) = −13.670, *P*< 0.01 by paired t test; n = 4), SBP (Saline: t (3) = −0.266, *P* = 0.808 by paired t test; muscimol: t (3) = 0, *P* = 1 by paired t test; Post: t (3) = −0.658, *P* = 0.557 by paired t test; n = 4), DBP (Saline: t (3) = 3.747, *P* < 0.05 by paired t test; muscimol: t (3) = 1.567, *P* = 0.215 by paired t test; Post: t (3) = 7.833, *P*< 0.01 by paired t test; n = 4), MAP (Saline: t (3) = 6.548, *P* < 0.01 by paired t test; muscimol: t (3) = 0.775, *P* = 0.495 by paired t test; Post: t (3) = 5.960, *P*< 0.01 by paired t test; n = 4) in anesthetized MI mice before, during, and after muscimol infusion into the MnR. Data are mean ± SEM. *P < 0.05; **P < 0.01, ***P < 0.001.

After MI, we observed that the anatomical association between M1 and MnR remained intact (Figure S6A, B, Supporting Information). To verify whether the M1‐MnR circuit was involved in the regulation of cardiac function in MI mice, we injected anterograde trans‐neuronal transport of AAV1‐CMV‐Cre‐GFP into the M1 and AAV9‐DIO‐ChR2‐mCherry into the MnR in MI mice (**Figure** **9**A). We found that photostimulation of the expressed ChR2 in MnR neurons postsynaptic to M1 significantly increased HR and the LF/HF power ratio (Figure 9B,C,D). The echocardiography results showed that activation of M1 neurons significantly reduced LVEF, and LVFS, increased LVIDs and SBP (Figure 9E,F,G,I,J), and decreased DBP and MAP (Figure 9K,L). There was no difference in LVIDd (Figure 9H). Moreover, we also expressed ChR2 in the M1 neurons and optical fibers were implanted above the MnR (Figure 9M). While optogenetic activation of the M1 neurons affected cardiac function and blood pressure in MI mice (Figure 9N–W), such effects were blocked by injection of muscimol into the MnR (Figure 9N–W). These functional results support that M1 neurons effect heart function in MI mice through projecting to the MnR. However, we did not find that the Ca^2+^ transients in the M1 neurons during ligation of the left anterior descending coronary artery (Figure S6C, D, Supporting Information).

## 3 Discussion

In clinical practice, acute MI often induces ventricular arrhythmia, such as ventricular tachycardia and ventricular fibrillation that is a risk factor for sudden cardiac death. Meanwhile, accumulating evidences show that intracranial hemorrhage and brain trauma lead to cardiac arrhythmias and cardiac dysfunction.^[^ ^6^, ^15^ ^]^ These facts prompt us to reveal the circuits and mechanisms of the brain regulation of cardiac functions. The cerebral cortex is involved in processing sensory information and motor function. Although studies have suggested an anatomical relationship between the cortex and heart function, the exact cause‐and‐effect relationship was still unclear.^[^ ^3^, ^9^ ^]^ In the present study, we identified a specific group of glutamatergic neurons in the M1, which project to MnR region and potentially affect sympathetic activity, HR, cardiac function, electrical conduction, and blood pressure in normal mice. Importantly, manipulation of M1 neuronal activity can also affect cardiac function in mice under the pathological conditions. These results update our understanding of the function of M1, and illustrate a novel target potentially important for the treatment of cardiac dysfunction in patients with acute MI.

Tracing with PRV is useful for establishing connectivity between the peripheral organs and key brain areas in the CNS. Ter Horst GJ, et al.^[^ ^3^ ^]^ injected PRV into the left ventricular myocardium of rat, and found PRV‐labeled neurons in the dorsal motor nucleus of (DMV), NTS, and Layer V of the dysgranular insular cortex in rats with spinal cord transection. This result suggests that the output of the cerebral cortex could regulate the parasympathetic control of the heart. By contrast, in the sympathetic pathway, PRV‐labeled neurons are in the sympathetic ganglia (SG), intermediolateral column (IML) in the spinal cord, RVLM and Layer V of the motor cortex.^[^ ^16^ ^]^ In this study, we confirmed the power of transneuronal tracing with PRV and unraveled the circuits from the heart to the cerebral cortex by injecting PRV into the left (PRV‐EGFP) and right (PRV‐mRFP) ventricular walls, and imaging with fMOST. As expected, the PRV labeled MnR, NTS, RVLM, MPOA, PVN, VMH, mPFC and cortical regions including M1, M2 and somatosensory cortex. Interestingly, ∼40% of the infected cortical neurons showed overlapping of EGFP with mRFP in the M1 (Figure 1). It is generally believed that cardiac innervation is asymmetrical.^[^ ^17^ ^]^ This is also demonstrated by the fact that neurons traced retrograde from the left and right ventricles do not coincide perfectly in M1.

The cerebral cortex is largely involved in processing sensory information and integrating higher motor function. Previous studies have found that electrical stimulation of the prefrontal cortex or insular cortex induced changes in HR. However, the underlying mechanism was not clear. In this study, we found that the HR, LVEF, and blood pressure of the normal mice were all increased after activating glutamatergic neurons (excitatory neurons) in the M1 region, and decreased when the activation of glutamatergic neurons was inhibited or ablated by inducing neurons apoptosis. These manifestations are similar to the activation or inhibition of sympathetic neural activity. We also found that activation of glutamatergic neurons in the M1 region increased the LF/HF power ratio, and inhibition or ablation of them reduced the ratio. These results confirmed that the M1 region could positively affect sympathetic nerve activation, which would increase HR, arterial contraction, arterial wall pressure, oxygen consumption and decrease myocardial reserve and coronary blood flow. Interestingly, ablation of M1 glutamatergic neurons increased the HW/BW ratio, and down‐regulated TH protein expression in the left ventricle. Based on these results, we speculated that in normal mice, manipulation of neuronal activity in M1 region disrupts the sympathovagal balance, especially regulates sympathetic nerve activation. Ablation of M1 glutamatergic neurons keeps HR in a low level of activity, which induces compensatory hypertrophy of heart to compensate for the decreased pumping function. It has been reported that the long‐term control of sympathetic nerve activity is associated with various cardiovascular diseases and increased mortality.^[^ ^18^ ^]^ Comprehending the neural activity in M1 region is important to regulate the cardiac function in physiological condition. It remains to obtain more evidence for explaining the similarities and differences of M1 in regulating sympathetic and parasympathetic neural activities.

The MnR is an important source of serotonergic innervations of the forebrain, and has been proved to project to sites which are involved in cardiovascular regulation. Blood pressure and HR are influenced by neuromodulation. Liberles et al reported that PIEZO2 neurons in the vagal and glossopharyngeal nerves can regulate baroreceptor reflex and aortic depressor nerve effects on blood pressure and HR.^[^ ^19^ ^]^ In our results, viral‐genetic labeling revealed the excitatory output from M1 neurons to the MnR. However, its role in HR regulation is still controversial. Electrical stimulation of the MnR increased the carotid blood pressure and the plasma levels of NE and adrenaline in the MnR.^[^ ^20^ ^]^ Local application of oxytocin or orexin microinjection in the MnR increased HR.^[^ ^21^ ^]^ Moreover, another report showed that HR was not affected after tetrodotoxin (a blocker of action potential) perfusion into the MnR.^[^ ^22^ ^]^ Similarly, electrical stimulation of the MnR was found to increase arterial blood pressure, but not in HR and respiratory rate.^[^ ^23^ ^]^ Both L‐glutamate microinjections or electrical stimulation of MnR evoked increase in blood pressure, while the effect on HR was negligible in urethane‐anesthetized male rats.^[^ ^24^ ^]^ Considering the different conditions of these experiments, the state of consciousness of mice (awake or anesthesia) would be important to confirm MnR function on HR. In the present study, we found that activating M1‐MnR pathway enhanced HR, LVEF and blood pressure. Thus, our results demonstrate a new pathway from M1 neurons directly to the MnR for affecting cardiac function.

Studies on cardiovascular disease have shown that multiple cardiovascular diseases lead to cardiac nerve remodeling, such as overactivity of sympathetic nerve and suppression of parasympathetic neural output, which increase the risk of sudden cardiac death.^[^ ^25^ ^]^ Histological studies also demonstrated that extensive alteration of sympathetic nerve endings in the peri‐infarcted area and autonomic dysfunction are an important factor for cardiac dysfunction in post‐infarction heart failure. However, little is known about the role and changes in neuronal activity in M1 region. In the present study, we found that activation of M1 glutamatergic neurons also increased the LF/HF power ratio in the mice with MI; by contrast, it reduced LVEF, LVFS, DBP, MAP, but increased LVIDs and SBP. These results indicate that activation of M1 also disrupts the stability of sympathetic and parasympathetic nerves. Considering the reduced myocardial contractility of the infarcted heart, increased HR may shorten the diastole period of the heart and reduce the amount of blood returning to the heart, thereby reducing cardiac function. In the present study, inhibiting the activity of glutamatergic neurons in the M1 region reduced HR and LF/HF power ratio, increased LVEF, LVFS, MAP and reduced LVIDs. Moreover, inducing M1 neurons apoptosis also reduced TH expression and NE level in the infarcted heart. It remains to detect the changes of activity of glutamatergic neurons in the M1 region after MI; however, down‐regulating their activity could increase cardiac function and reduce the expression of collagen‐1 and collagen‐3 proteins. The reason could be related to the reversal of sympathetic overactivation, which reduces excessive HR and increases cardiac blood volume. It has also been reported that targeting the central nervous system to prevent sympathetic overactivation inactivated the development of cardiac fibrosis in animal models of heart failure,^[^ ^26^ ^]^ which supports our discovery. Furthermore, after MI, there were still fibrous projections from M1 to the MnR. But there was no significant change in calcium activity in M1 neurons of MI mice. Thus, it maybe only affecting the efferent nerves of the heart but not the afferent nerves. Activation of glutamatergic neurons projecting from the M1 to the MnR caused an increase in HR and a decrease in cardiac function, and this effect were blocked by muscimol from the MnR. Thus, the M1‐MnR circuit could be involved in the effect of cardiac function after MI. Therefore, our results demonstrate that the M1 glutamatergic neurons may be an essential central area regulating cardiac function and the balance of autonomic nerves in MI mice.

These findings are important for understanding the neural mechanisms underlying cardiovascular regulation and provide new therapeutic strategies for MI. However, it remains to investigate neural projections from the M1 to other brain regions that also regulate cardiac function and blood pressure as well as potential differences in the effects of bilateral M1 on the regulation of cardiac function and blood pressure. It is also interesting to clarify whether the target of M1 affecting autonomic nerve balance is sympathetic or parasympathetic nerves. Furthermore, it will be important to study the relationship between brain activities and heart function at awake state. As a potential therapeutic target site accessible to brain stimulation methods in treating sympathetic hyperactivation after MI, further studies on M1‐associated cardiovascular regulation of neural circuits is warranted.

## 4 Experimental Section

### Animals

The Care and experimental manipulations of all animals were conducted in accordance with the NIH Guide for Care and Use of Laboratory Animals, and all the protocols and procedures were approved by the Institutional Animal Care Use Committee at the Shaanxi Normal University. Adult male C57BL/6 mice (8–12 weeks) were purchased from the Laboratory Animal Center of Xi'an Jiaotong University. All mice were maintained under a reversed 12‐h light/dark cycle with ad libitum access to water and food. During the experiment, mice were continuously ventilated with a mixture of 1.5–2% isoflurane and O_2_ until the end of the experiment. After the experiment, mice were anesthetized with 5% isoflurane and euthanized by bloodletting. For all measurements, a minimal depth of anesthesia was strictly maintained at equivalent levels between each group of mice as determined by a breathing rate of 90–110 breaths per minute combined and the loss of the “toe pinch‐pedal reflex.”

### MI Surgery

MI model was established by permanent ligation of anterior descending branch of the left coronary artery. The ligation site was 1–2 mm below the junction of the left atrial appendage and pulmonary conus. Surface electrocardiography (ECG) was recorded using the PowerLab data acquisition system (AD Instruments, New South Wales, Australia) throughout the procedure. The success of the MI model was confirmed by observing ST‐segment elevation or T‐wave inversion on the surface ECG after ligation. To ensure the consistency in model construction, all MI operations were performed by the same experienced individual.

### Echocardiography

Mice were lightly anesthetized with 1.5–2% isoflurane inhalation before undergoing two‐dimensional and M‐mode echocardiography in the parasternal long‐axis view. Echocardiographic parameters, including LVEF, LVFS, and LVIDs and LVIDd were obtained by using a Doppler ultrasound instrument (VINNO 6 VET, VINNO, China). Three consecutive cardiac cycles were used for all the measurements. Body temperature was maintained in the physiological range (36–37.5 °C) throughout the procedure using a heating pad.

### Heart Rate Variability (HRV) Analysis

During the experiment, all mice were anesthetized with 1.5–2% isoflurane. The surface ECG signal was recorded for 10 min using the PowerLab data acquisition system. A 5‐min ECG segment with a stable signal was selected and analyzed.^[^ ^27^ ^]^ The analysis was performed on 2‐min bins before and after optogenetic stimulation.^[^ ^28^ ^]^ The LabChart software (version 8.0, AD Instruments) was used to analyze HRV and determine the frequency‐domain indexes of HRV, including HF power (1.5‐5 Hz, which indicates parasympathetic tone),LF power (0.15–1.5 Hz, which possibly correlates with sympathetic tone). The LF/HF power ratio was calculated to reflect the balance between sympathetic and vagal tones.

### Blood Pressure Test

Tail arterial blood pressure during the conscious state (chemogenetic or ablating M1 excitatory neurons) was measured using a non‐invasive blood pressure system (Kent Scientific). During optogenetic experiments, blood pressure was monitored under anesthesia. The core body temperature was maintained at 36.5 ± 0.5 °C using a heating pad. DBP, SBP and MAP were measured.

### PRV Retrograde Tracing from the Heart

Mice were lightly anesthetized by continuously inhaling 1.5–2% isoflurane. The left intercostal spaces of mice were opened between the third and fourth ribs. The heart was extruded from the intercostal space. The retrograde trans‐polysynaptic PRV was injected into the left ventricular wall (PRV‐CAG‐EGFP, 1 µl, 2.00 × 10^9^ PFU mL^−1^) or right ventricular wall (PRV‐CAG‐mRFP, 1 µl, 2.00 × 10^9^ PFU mL^−1^) at different sites respectively via a glass micropipette. After ≈132 h (5.5 d), mice were anesthetized using 5% isoflurane, and the heart and brain were rapidly collected. The accuracy of the injection was determined for each animal.

### Stereotactic Surgeries and Intracranial Injection

Male mice, 8 weeks‐old, were lightly anesthetized with 1.5‐2% isoflurane and placed in a stereotaxic apparatus (RWD Life Science Co., Ltd. Shenzhen, China). The coordinates used were based on the Paxios and Franklin's Mouse Brain Atlas (2nd edition), which included bilateral M1 region (AP, −0.70 mm/−0.90 mm/−1.10 mm, ML, ±1.00 mm, DV, −0.58 mm), M2 region (AP, −0.90 mm, ML, ±0.8 mm, DV, −0.58 mm), S1 region (AP, −0.90 mm, ML, ±1.2 mm, DV, −0.58 mm) and MnR region (AP, −4.36 mm, ML, ±0.00 mm, DV, −4.8 mm).

For optogenetics, mice were injected with AAV2/9‐CaMKIIα‐mCherry (50 nl, titer 2.70 × 10^12^ vg ml^−1^) or AAV2/9‐CaMKIIα‐ChR2‐mCherry (50 nl, titer 2.53 × 10^12^ vg ml^−1^) into the bilateral M1. Alternatively, AAV1‐CMV‐Cre‐GFP (50 nl, titer 1.19 × 10^12^ vg ml^−1^) was injected into the M1 region, and AAV9‐DIO‐ChR2‐mCherry (50 nl, titer 2.52 × 10^12^ vg ml^−1^) was injected into the MnR region.

For chemogenetic, AAV2/9‐CaMKIIα‐mCherry (50 nl, titer 2.70 × 10^12^ vg ml^−1^) or AAV2/9‐CaMKIIα‐hM4Di‐mCherry (50 nl, titer 2.41 × 10^12^ vg ml^−1^) was injected into the bilateral M1 region.

To ablate neurons in M1 region, mice were microinjected AAV2/8‐CaMKIIα‐Cre‐GFP (40 nl, titer 2.93 × 10^12^ vg ml^−1^) and AAV2/9‐EF1α‐DIO‐taCasp3‐TEVp (40 nl, titer 2.24 × 10^12^ vg ml^−1^) into the bilateral M1 region. The mice of control group were only injected with AAV2/9‐EF1α‐DIO‐taCasp3‐TEVp (80 nl, titer 2.24 × 10^12^ vg ml^−1^).

To conduct optogenetics and fiber photometry, AAV2/1‐hSyn‐Cre (50 nl, titer 1.09 × 10^13^ vg ml^−1^) and AAV2/9‐EF1α‐DIO‐ChR2‐mCherry (50 nl, titer 2.52 × 10^12^ vg ml^−1^) were delivered into the bilateral M1. Additionally, AAV2/9‐EF1α‐DIO‐GCaMP6s (50 nl, titer 2.59 × 10^12^ vg ml^−1^) was injected into the MnR. AAV2/9‐hSyn‐GCaMp6f (80 nl, titer 2.58 × 10^12^ vg ml^−1^) for fiber photometry.

For optogenetic and pharmacology experiments, AAV2/9‐CaMKIIα‐ChR2‐mCherry (50 nl, titer 2.53 × 10^12^ vg ml^−1^) was injected into the bilateral M1 (AP, −0.90 mm; ML, ±1.00 mm; DV, −0.58 mm). After four weeks, optical fibers were implanted above the bilateral M1. The cannula was stereotaxically implanted at the MnR. Post‐operative recovery lasted for five days, after which muscimol (0.5 µg µl^−1^ in ACSF, G019, Sigma) was injected into the MnR. Optogenetic stimulation was carried out 10 min later. CTB555 (CTB‐01, BrainVTA, Wuhan, China) was delivered into the MnR after completing all experiments.

To identify the outputs of M1 neurons, H129‐hUbC‐EGFP (50 nl, titer 1.2×10^9^ PFU/mL), VSV‐mCherry (50 nl, titer 2.00 × 10^9^ IFU/ml) or AAV2/9‐CaMKIIα‐EGFP (50 nl, titer 2.08 × 10^12^ vg ml^−1^) were injected into bilateral M1. Histological analysis was conducted one week after VSV‐EGFP injection and four weeks after AAV2/9‐CaMKIIα‐EGFP injection.

All viruses and muscimol were delivered into the brain regions at a rate of 50 nl min^−1^ using a microsyringe pump (KD Scientific, MA, USA). To prevent backflow, the pipette was kept in place 10 min after injection. All viruses used were purchased from BrainVTA.

### Fluorescent Micro‐Optical Sectioning Tomography (fMOST) Imaging

Whole brain images were carried out by using the fMOST‐5000 system.^[^ ^29^ ^]^ Briefly, the brain was embedded in resin and immersed in 0.05 mol/L Na_2_CO_3_ during the imaging process. The fMOST system automatic sectioned with 1 µm steps and imaged at a 0.35 × 0.35 × 1 µm voxel sampling rate using two channels (EGFP‐labeled neurons and mRFP‐labeled neurons) in 16‐bit depth. The visualized data were analyzed using Amira software and Imaris software.

### Optogenetics

Three or four weeks after virus injection, mice were implanted with optical fibers (OD = 250 mm, 0.37 NA; Shanghai Fiblaser) in the bilateral M1 (AP, −1.10 mm; ML, ±1.14 mm; DV, −0.58 mm. DV: 10° angle), M2 (AP, −0.9 mm; ML, ±0.99 mm; DV, −0.6 mm. DV: 10° angle), and S1 (AP, −0.9 mm; ML, ±1.39 mm; DV, −0.6 mm. DV: 10° angle). After seven days, optogenetic stimulation was performed as previously described.^[^ ^30^ ^]^ Subjects received stimulation approximately 10 mW mm^−2^ for 15s (473 nm, 20 Hz, 15 ms pulses). Cardiac function and blood pressure were recorded at baseline and post‐photostimulation.

### Fiber Photometry

Multi‐channel fiber was used photometry system (Thinker Tech Nanjing Biotech Co., Ltd. Nanjing, China) to detect GCaMP6m fluorescent signals as described previously.^[^ ^31^ ^]^ Briefly, modulated blue 480 LEDs were reflected by dichroic mirrors, focused though a ×10 objective lens (NA = 0.3; Olympus) and an optical commutator was coupled to it. The blue light was guided between the commutator and the implanted optical fiber. The optical fiber patch cord (230 µm O.D., NA = 0.37, 2‐m long) was connected to a multi‐channel fiber photometry system. The implanted optical fiber, which measured 5 mm in length for the MnR region, and 2 mm in length for the M1 region, was securely attached to a custom patch cord using a ferrule. For MnR GCaMP6m signals, M1 region was given blue light stimulation (473 nm, 20 Hz, 15 ms pulses, 10 s on 2 s off cycle). For M1 GCaMP6m signals, Ca^2+^ transients were recorded uring acute ligation of the left anterior descending coronary artery or sham. The recording data were analyzed by the MATLAB2017b. Then, the delta F/F was calculated as *∆*F/F  =  (F − F0)/F0.

### Chemogenetic

CNO (BrainVTA) was intraperitoneally administered at a dosage of 1 mg k^−1^g, 30 min prior to cardiac function and blood pressure test for modulating M1 neurons. In MI mice, CNO was injected intraperitoneally once a day for 28 days for chronic modulation of M1 neurons expressing the inhibitory designer receptor exclusively activated by designer drugs receptor, hM4Di. Cardiac function and blood pressure were measured in MI mice 30 min after injecting CNO on day 28. In all control groups, the mice were injected with the same amount of saline as CNO.

### Ablating M1 Excitatory Neurons

To ablate M1 neurons, AAV2/8‐CaMKIIα‐Cre‐GFP and AAV2/9‐EF1α‐DIO‐taCasp3‐TEVp were injected into the bilateral M1 of mice. The mice of control group were injected only with AAV2/9‐EF1α‐DIO‐taCasp3‐TEVp. After four weeks of virus expression, mice were lightly anesthetized with 1.5–2% isoflurane and then, cardiac function and blood pressure were detected. After that, mice were anesthetized with 5% isoflurane and sacrificed, the heart was dissected out and fixed in pre‐cooled 4% paraformaldehyde (PFA) or liquid nitrogen for subsequent tests.

### Immunohistochemistry

After being anesthetized with 5% isoflurane, mice were transcranial perfused with 0.01 M phosphate‐buffered saline (PBS) followed by 4% PFA. Then, the brains were dissected and post‐fixed in newly prepared 4% PFA solution for approximately three days before being sunk in 30% sucrose. To visualize viral expression and fiber placement, 40 µm‐thick sections were cut using the cryostat (CryoStar NX50 OPD, Thermo Scientific). The brain sections were washed with 0.01 M PBS for 10 min to remove optimal cutting temperature compound. Then, the sections were permeabilized with 0.3% Triton X‐100 in 0.01 M PBS for 20 min and blocked with normal goat serum albumin for 30 min at room temperature before incubating with primary antibodies overnight at 4 °C. The primary antibodies used were as follows: rabbit primary antibody against neuron‐specific nuclear protein (NeuN, a mature neuronal marker, Abcam, ab177487, 1:4000), goat primary antibody against TPH2 (Abcam, ab121013, 1:500), mouse monoclonal antibody against VGlut2 (Abcam, ab79157, 1:1000) and rabbit primary antibody against c‐Fos (Abcam, ab190289, 1:2000). At the following day, unbound primary antibodies were removed by washing three times in 0.01 M PBS, each for 5 min. Staining was developed by using goat anti‐rabbit conjugated with TIRTC (Jackson ImmunoResearch, Cat# 111‐025‐003, 1:200) and AlexaFluor 647 affinipure goat anti‐mouse (Jackson ImmunoResearch Cat# 115‐605‐003, 1:200), donkey anti‐goat conjugated with AlexaFluor 488 (Jackson ImmunoResearch, Cat# 705‐545‐1471, 1:200) or goat anti‐rabbit conjugated with Alexa fluor488 (Jackson ImmunoResearch, Cat# 111‐545‐003, 1:200) for 1 h at room temperature. All brain sections were counterstained with 4′,6‐diamidine‐2′‐phenylindole dihydrochloride (DAPI) (1 µg/ml, Boster, AR1177) for 10 min and washed with 0.01 M PBS three times, each for 5 min. Images were captured using a fluorescent microscope (Nikon, 55i).

### Cardiac Tissue Collection and Histological Staining

Myocardial tissue from the left ventricle of mice was harvested, rinsed with saline, drained, and weighed. The heart tissue was fixed in 4% PFA for histological staining or frozen in liquid nitrogen for protein analysis. Body weight (BW) and heart weight (HW) were measured to calculate HW/BW for the evaluation of cardiac hypertrophy. Heart samples were fixed in 4% PFA for 72 h and embedded in paraffin. The slices with a thickness of 5 µm were prepared and stained with Masson's trichrome. The infarction level was analyzed using Image Pro Plus analysis software and expressed as the volume fraction of collagen (CVF % = collagen area/total tissue area ×100%).

### Enzyme‐Linked immunosorbent assay (ELISA)

To measure sympathetic nervous activity, the concentration of the sympathetic transmitter NE in the left ventricular myocardium and the blood and was detected. Two mL blood sample was collected via the arterial catheter from the mice under anesthesia with 5% isoflurane at the end of the experiment. The blood sample was then centrifuged at 3,000 rpm for 10 min and the serum was collected. Serum and cardiac level of NE was quantified using a NE kits (Elabscience, E‐EL‐0047c) and assessed with a biochemical analyzer in accordance with the kit instructions.

### Western Blotting

Myocardial tissues from left ventricle or peri‐infarcted area were homogenized with RIPA lysis buffer containing PMSF and protease inhibitor. The protein concentration was quantified by using a BCA Protein Assay Kit (PQ003, Shaanxi ZhongHuiHeCai bio‐pharmaceutical Technology Co., Ltd. Xi'an, China), and diluted to a final protein concentration of 5 µg µl^−1^. Proteins were transferred onto polyvinylidene fluoride (PVDF) membranes and incubated with 5% blocking buffer (Bovine Serum Albumin, BSA) for 2 h at room temperature. The membranes were incubated with the following diluted primary antibodies overnight at 4 °C: tTH (a rate‐limiting enzyme in NE synthesis; Abcam, ab112, 1: 1000), glyceraldehyde‐3‐phosphate dehydrogenase (GAPDH) (Genetex, GTX100118, 1: 5000), collagen‐1 (Genetex, GTX26308, 1:1000) or collagen‐3 (Genetex, GTX637655, 1: 500). At the following day, after washing with 1 × Tris‐Buffered Saline Tween‐20 (TBST), the membranes were incubated with the secondary antibodies which were diluted in 5% milk. The protein bands were detected with enhanced chemiluminescence (Bio‐Rad, USA) and the Image Systems (Bio‐Rad, USA).

### Statistical Analysis

All parameters were measured by the same experienced individual who was blinded to the group information. All data were acquired and analyzed offline by a single blinded observer. The data were presented as mean ± SEM and analyzed using SPSS 22.0. Normality of all data were assessed using the Kolmogorov‐Smirnov test. Optogenetics [treatment (mCherry/ChR2) × stimulus (off/on)] and chemogenetics [treatment (mCherry/hM4Di) × stimulus (saline/CNO)] were analyzed by two‐way ANOVA with repeated‐measures followed by Sidak's multiple comparison post hoc test. If the data exhibited heterogeneity of variance, the two‐tailed non‐parametric tests were used. Independent t test (two‐tailed) was used to compare heart function and blood pressure between the control and taCasp3 groups, as well as c‐Fos's expression in ChR2/mCherry and hM4Di/mCherry. Additionally, a combination of optogenetics and Ca^2+^ signal recording was analyzed by one‐way ANOVA followed by Tukey's multiple comparison post hoc test. Optogenetics (M1‐MnR pathway), optogenetics and pharmacology, Ca^2+^ signals recording in the MI processing were tested with Paired t tests (two‐tailed). Statistical significance was determined at a P‐value of less than 0.05.

## Conflict of Interest

The authors declare no conflict of interest.

## Author Contributions

W.B. and M.C. contributed equally to this work. Z.T. and Z.H. conception and design of research; W.B., Z.H., M.C., Y.M., L.D., Y.G., H.L., C.T. performed experiments; W.B., Z.H., Y.M., L.D. analyzed data; W.B., Z.H., Z.T. prepared figures; W.B., Z.H., M.C. drafted the manuscript; Z.T., W.B., Z.H., M.C., F.T. edited and revised the manuscript. All authors have read and approved the final version of the manuscript. The authors thank Yufeng Wang (Harbin Medical University and Zhejiang Chinese Medical University), Larry J Young (Emory University) and Xinming Ma (University of Connecticut) for their insightful comments on this study and extensive editing of the manuscript.

## Supporting information

Supporting Information

Supplementary Video S1

## Data Availability

The data that support the findings of this study are available in the supplementary material of this article.
