# Supplementary material for: Manipulation of Glutamatergic Neuronal Activity in the Primary Motor Cortex Regulates Cardiac Function in Normal and Myocardial Infarction Mice
Source: Adv Sci (Weinh). 2024 Mar 15;11(20):2305581. doi: 10.1002/advs.202305581 (PMC11132081; doi:10.1002/advs.202305581)
Supplement: Supplementary file 1 — Supporting Information [file ADVS-11-2305581-s001.pdf]

ADVANCED SCIENCE
Open Access

Supporting Information

for *Adv. Sci.*, DOI 10.1002/advs.202305581

Manipulation of Glutamatergic Neuronal Activity in the Primary Motor Cortex Regulates Cardiac Function in Normal and Myocardial Infarction Mice

Wenyan Bo, Mengxin Cai, Yixuan Ma, Lingyun Di, Yanbin Geng, Hangzhuo Li, Caicai Tang, Fadao Tai, Zhixiong He and Zhenjun Tian**

Supplementary Information for *Manipulation of Glutamatergic Neuronal Activity in the Primary Motor Cortex Regulates Cardiac Function in Normal and Myocardial Infarction Mice*

Wenyan Bo#, Mengxin Cai#, Yixuan Ma, Lingyun Di, Yanbin Geng, Hangzhuo Li, Caicai Tang, Fadao Tai, Zhixiong He* and Zhenjun Tian*

Figures. S1 to S6.

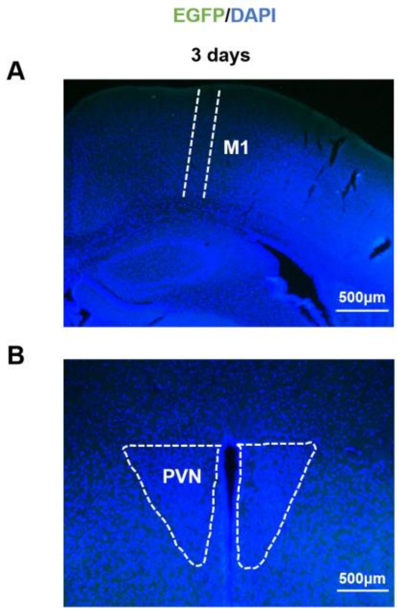

Figure. S1 Image of brain which injected PRV in heart after 3 days

A, B) Representative images showing PRV-infected neurons in different brain areas at 3 days after the viral injection. Scale bar, 500 μm.

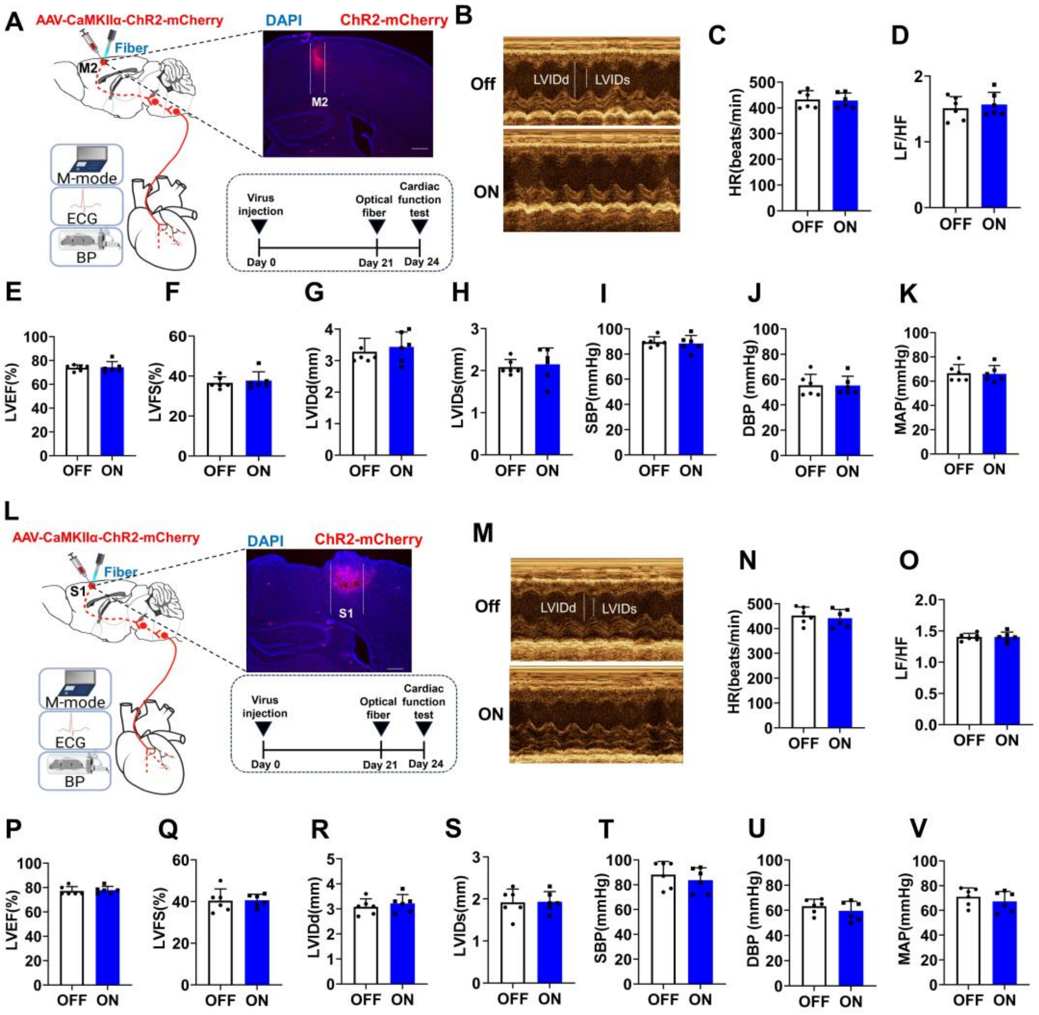

Figure. S2 Activation of glutamatergic neurons in M2 and S1 has no effect on heart function and blood pressure in normal mice

A) Experimental schematics and a representative image showing ChR2-mCherry expression in M2. Scale bar, 200 μm. B) Examples of echocardiographic images in anesthetized mice. C, D, E, F, G, H, I, G, K) HR (t (5) =0.937, P =0.292 by paired t test; n= 6), LF/HF (t (5) =-0.958, P =0.382 by paired t test; n= 6), LVEF (t (5) = -0.214, P =0.839 by paired t test; n= 6), LVFS (t (5) = -0.592, P =0.580 by paired t test; n= 6), LVIDd (t (5) =-1.031, P =0.350 by paired t test; n= 6), LVIDs (t (5) =-0.598, P =0.576 by paired t test; n= 6), SBP (t (5) = 0.848, P =0.435 by paired t test; n= 6), DBP (t (5) = 0.191, P =0.856 by paired t test; n= 6), MAP (t (5) =1, P =0.363 by paired t test; n= 6). Data are mean ± SEM. *$P < 0.05$; **$P < 0.01$, ***$P < 0.001$. L) Experimental schematics and a representative image showing ChR2-mCherry expression in S1. Scale bar, 200 μm. M) Examples of echocardiographic images in anesthetized mice. N, O, P, Q, R, S, T, U, V) HR (t (5) =1.595, P =0.172 by paired t test; n= 6), LF/HF (t (5) =0.004, P =0.997 by paired t test; n= 6), LVEF (t (5) = -0.531, P =0.618 by paired t test; n= 6), LVFS (t (5) = -0.106, P =0.920 by paired t test; n= 6), LVIDd (t (5) =0.655, P =0.542 by paired t test; n= 6), LVIDs (t (5) = -3.07, P =0.771 by paired t test; n= 6), SBP (t (5) = 1.939, P =0.110 by paired t test; n= 6), DBP (t (5) = 1.870, P =0.120 by paired t test; n= 6), MAP (t (5) = 1.959, P =0.107 by paired t test; n= 6). Data are mean ± SEM. *$P < 0.05$; **$P < 0.01$, ***$P < 0.001$.

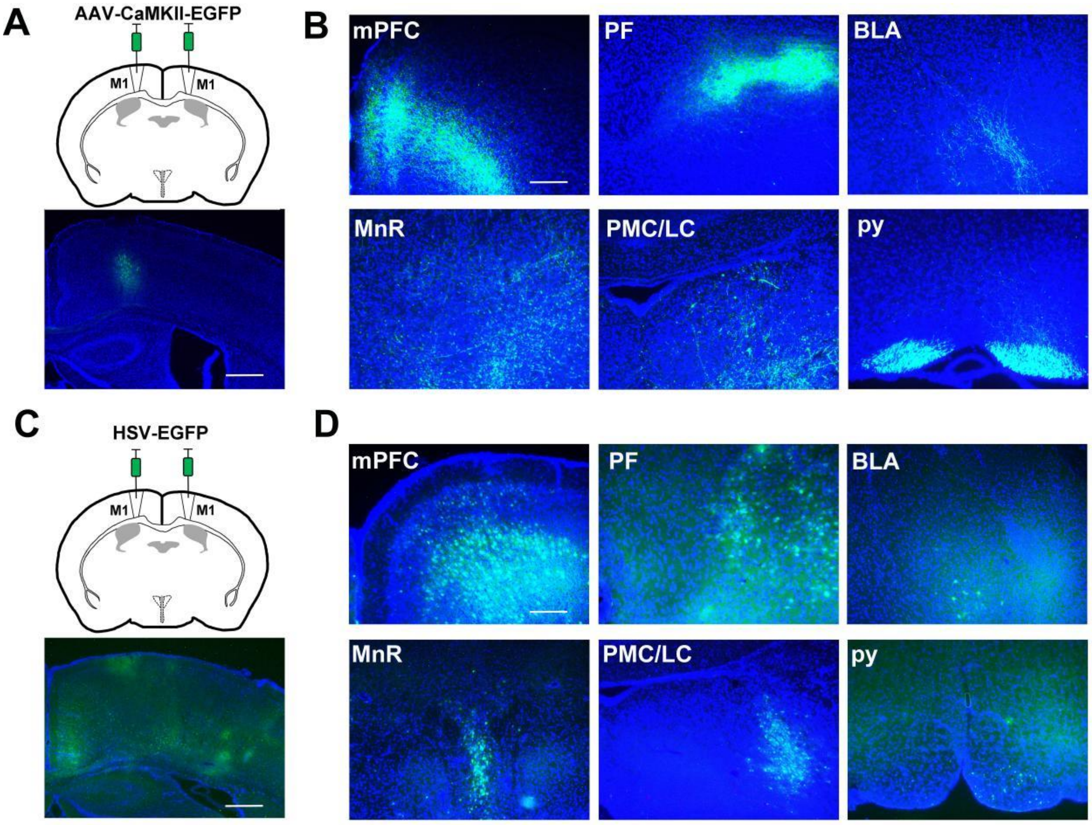

Figure. S3 Outputs of the M1 neurons

A) Schematics illustration of non-trans-synaptic anterograde tracing strategy. Scale bar, 500 μm. B) The M1 neurons project to the different brain regions after AAV-CaMKII-EGFP injection. Scale bar, 100 μm. C) Schematics illustration of trans-synaptic anterograde tracing strategy. Scale bar, 500 μm. D) Representative images of anterogradely labeled neurons in various brain areas. Scale bar, 100μm. mPFC, medial prefrontal cortex; PF, parafascicular thalamic nucleus; BLA, basolateral amygdaloid nucleus; MnR, median raphe nucleus; PMC, pontine micturition center; LC, locus coeruleus; py, pyramidal tract.

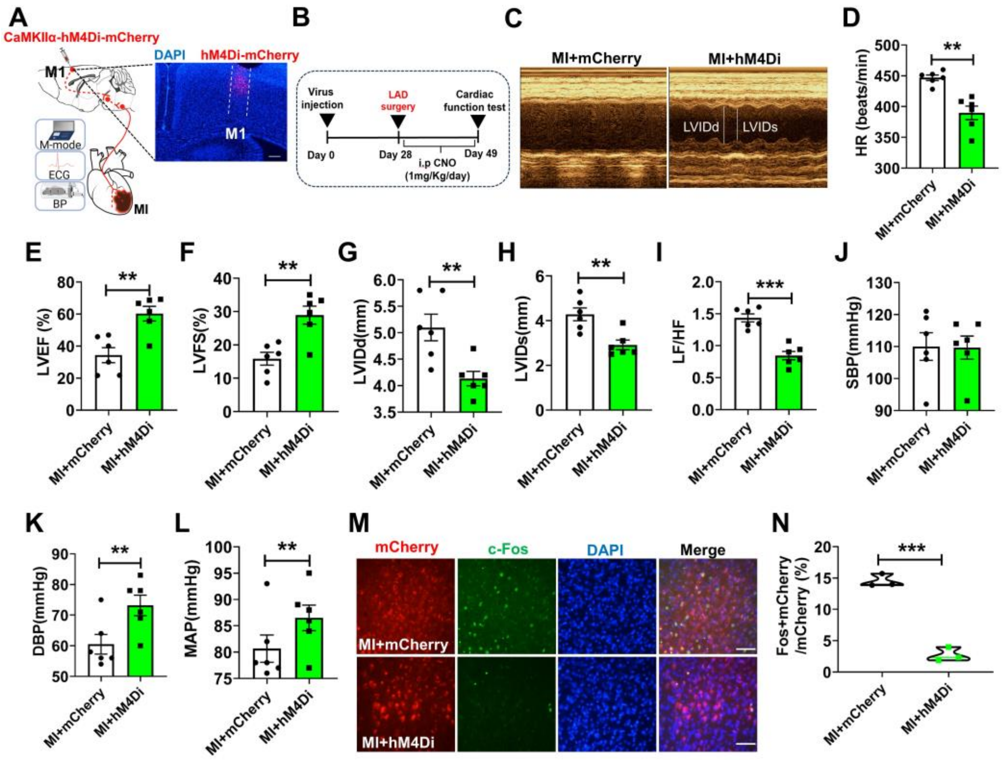

Figure. S4 Chemogenetic inhibition of M1 glutamatergic neurons regulated heart function in MI mice

A) Experimental schematics and a representative image showing hM4Di-mCherry expression in M1. Scale bar, 500μm. B) The experimental timelines. C) Examples of echocardiographic images in anesthetized mice. D-I) Quantification of HR ($t(10) = 2.634$, $P < 0.01$ by independent t-tests; MI+mCherry = 6, MI+hM4Di = 6), LVEF ($t(10) = -3.960$, $P < 0.01$ by independent t-tests; MI+mCherry = 6, MI+hM4Di = 6), LVFS ($t(10) = -3.956$, $P < 0.01$ by independent t-tests; MI+mCherry = 6, MI+hM4Di = 6), LVIDd ($t(10) = 3.394$, $P < 0.001$ by independent t-tests; MI+mCherry = 6, MI+hM4Di = 6), LVIDs ($t(10) = 3.821$, $P < 0.01$ by independent t-tests; MI+mCherry = 6, MI+hM4Di = 6) and LF/HF power ratio ($t(10) = 6.545$, $P < 0.001$ by independent t-tests; MI+mCherry = 6, MI+hM4Di = 6) in anesthetized mice. J-L) SBP ($t(10) = 0.059$, $P > 0.05$ by independent t-tests; MI+Dio = 6, MI+taCasp3 = 6), DBP ($t(10) = -3.816$, $P < 0.01$ by independent t-tests; MI+mCherry = 6, MI+hM4Di = 6) and MAP ($t(10) = -3.558$, $P < 0.01$ by independent t-tests; MI+mCherry = 6, MI+hM4Di = 6) in conscious mice. M) Immunohistochemical images showing overlap of hM4Di-mCherry (red) and c-Fos (green) positive neurons in the M1. Scale bar, 100μm. N) Quantification of c-Fos positive cells in the M1 (Independent t test. $t(4) = 13.490$, $P < 0.001$). Data are mean ± SEM. *$P < 0.05$; **$P < 0.01$, ***$P < 0.001$.

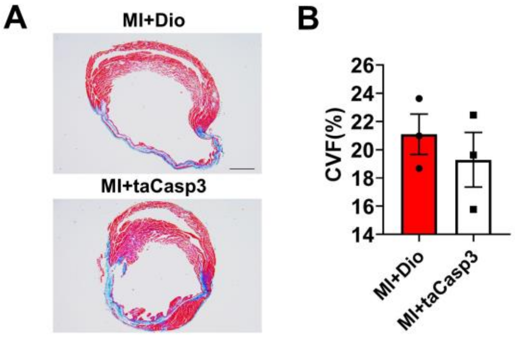

Figure. S5 Effect of M1 neurons ablation on cardiac collagen fibers in MI mice

A) Representative images showing myocardial collagen fiber staining, myocardial collagen fibers in blue and cytoplasm in red. B) Percentage of myocardial collagen fibers (CVF%) (t (4) = 6.531, $P < 0.01$ by independent t-tests.; MI+Dio = 3, MI+taCasp3 = 3).

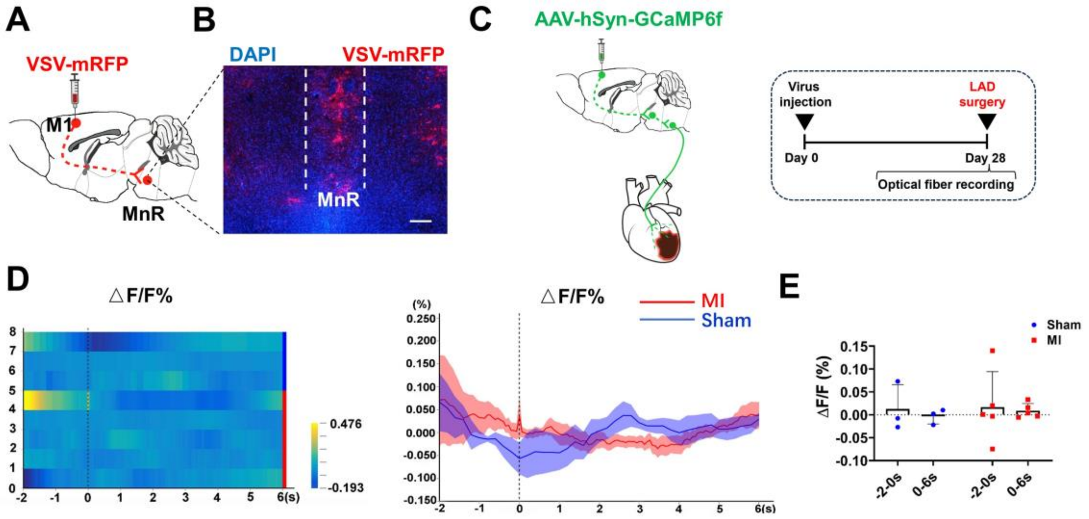

Figure. S6 The M1-MnR anatomical connection and cardiac function-associated Ca^{2+} transients in M1 neurons of MI mice

A) Schematics illustration of trans-synaptic anterograde tracing strategy. B) The M1 neurons project to MnR after VSV-mRFP injection. Scale bar, 500μm. C) Experimental schematics. D) Heatmap showing fluorescence signals (left). A representative trace of calcium signals recorded in M1 (right). E) Quantification of calcium signals (Sham: t (2) = 0.399, P =0.728 by Paired t test, n = 3; MI: t (4) = 0.247, P=0.817 by Paired t test. n = 5). Data are mean ± SEM. *$P < 0.05$; **$P < 0.01$.
